# Supplementary figures and images for: Soil, senescence and exudate utilisation: characterisation of the Paragon var. spring bread wheat root microbiome
Source: Environ Microbiome. 2021 Jun 21;16:12. doi: 10.1186/s40793-021-00381-2 (PMC8215762; doi:10.1186/s40793-021-00381-2)

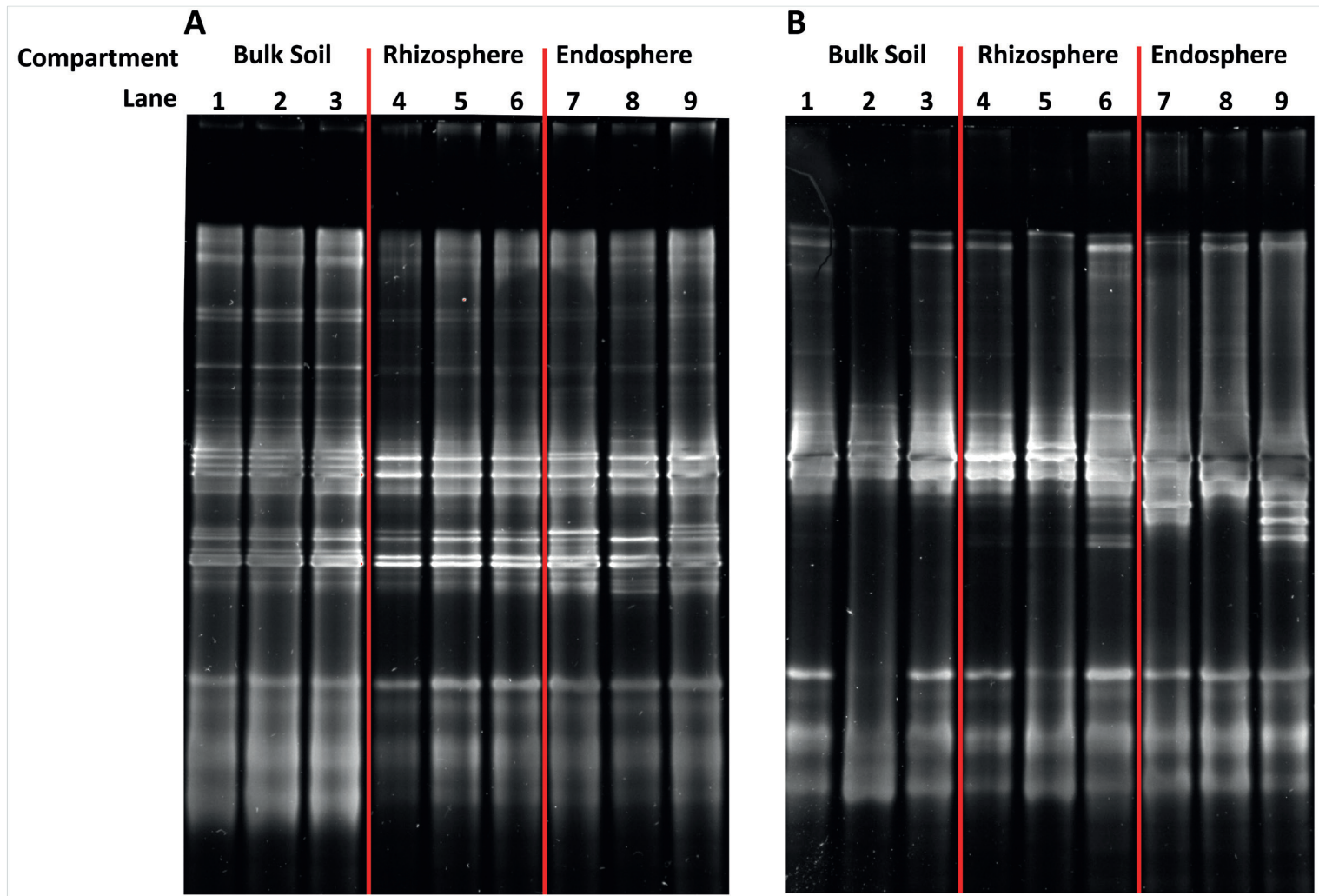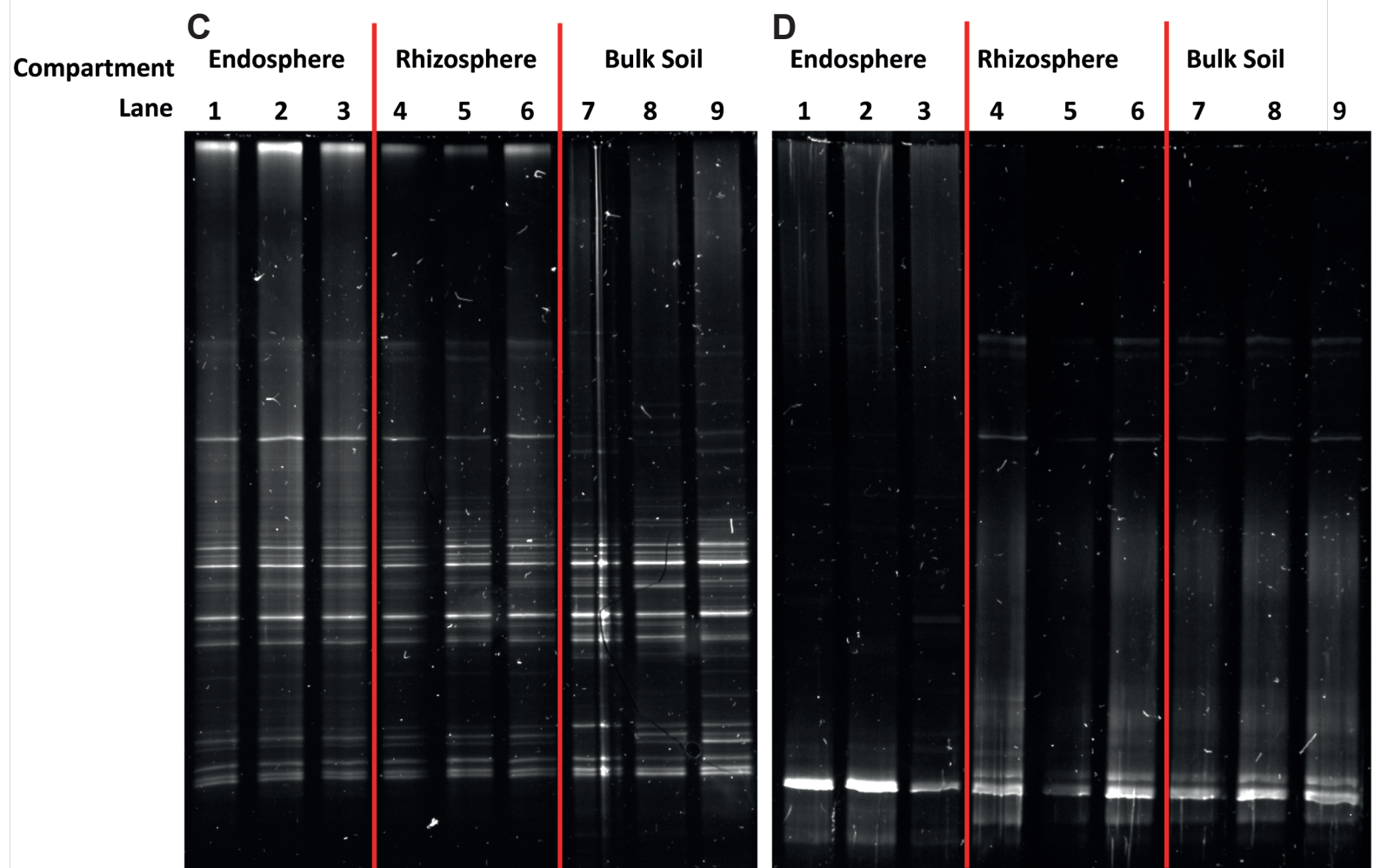

Supplement: Supplementary file 1 — Additional file 1: Supplementary Figure 1. Denaturing gel gradient electrophoresis (DGGE) showing archaeal 16S rRNA gene (A, B) or amoA (C, D) diversity across the bulk soil, rhizosphere and endosphere of wheat grown under laboratory conditions in agricultural soil (A, C) or Levington F2 compost (B, D). Primers are indicated in the Supplementary Table 6. [file 40793_2021_381_MOESM1_ESM.pdf]

Rhizosphere A

12C

| 1 | 2 | 3 | 4 | 5 | 6 | 7 | 8 | 9 | 10 | 11 | 12 |
|---|---|---|---|---|---|---|---|---|----|----|----|
|---|---|---|---|---|---|---|---|---|----|----|----|

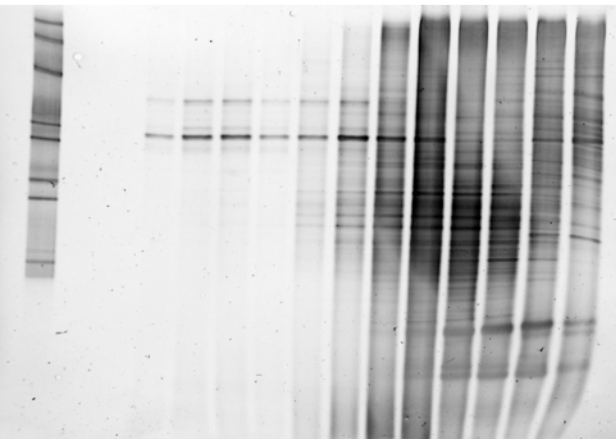

13C

| 1 | 2 | 3 | 4 | 5 | 6 | 7 | 8 | 9 | 10 | 11 | 12 |
|---|---|---|---|---|---|---|---|---|----|----|----|
|---|---|---|---|---|---|---|---|---|----|----|----|

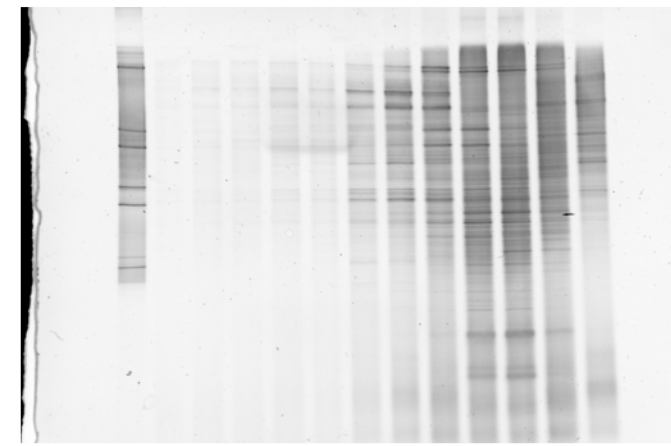

Rhizosphere B

12C

| 1 | 2 | 3 | 4 | 5 | 6 | 7 | 8 | 9 | 10 | 11 | 12 |
|---|---|---|---|---|---|---|---|---|----|----|----|
|---|---|---|---|---|---|---|---|---|----|----|----|

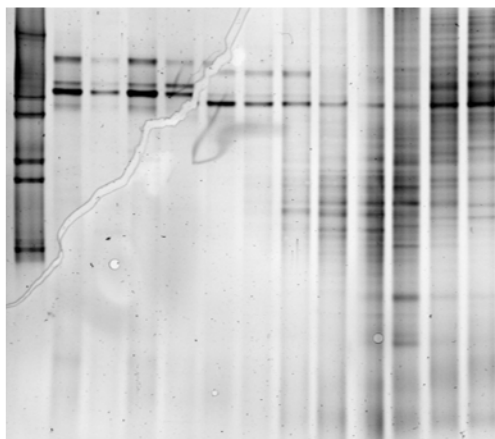

13C

| 1 | 2 | 3 | 4 | 5 | 6 | 7 | 8 | 9 | 10 | 11 | 12 |
|---|---|---|---|---|---|---|---|---|----|----|----|
|---|---|---|---|---|---|---|---|---|----|----|----|

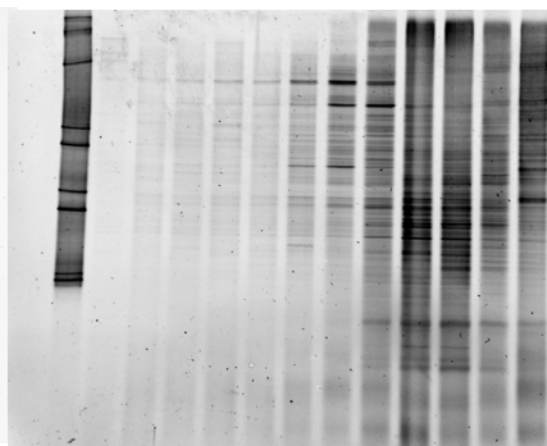

Rhizosphere C

12C

| 1 | 2 | 3 | 4 | 5 | 6 | 7 | 8 | 9 | 10 | 11 | 12 |
|---|---|---|---|---|---|---|---|---|----|----|----|
|---|---|---|---|---|---|---|---|---|----|----|----|

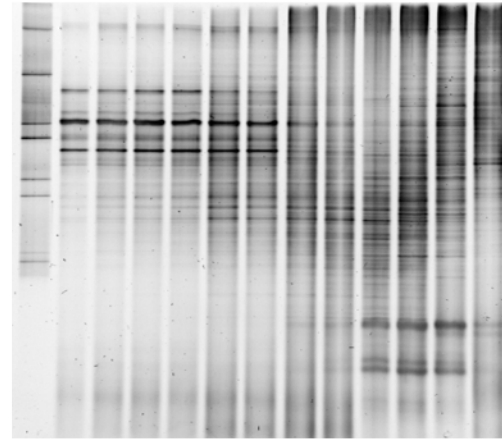

13C

| 1 | 2 | 3 | 4 | 5 | 6 | 7 | 8 | 9 | 10 | 11 | 12 |
|---|---|---|---|---|---|---|---|---|----|----|----|
|---|---|---|---|---|---|---|---|---|----|----|----|

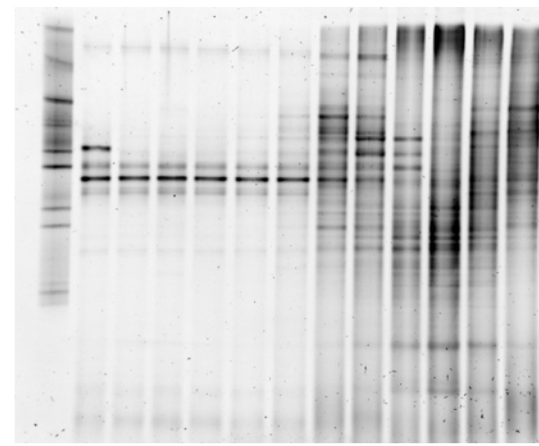

Supplement: Supplementary file 2 — Additional file 2: Supplementary Figure 2. Denaturing gel gradient electrophoresis (DGGE) showing bacterial 16S rRNA gene diversity across the 12 fractions generated for stable isotope probing for the rhizosphere associated with the 12C control (top) and 13C labelled (top) plant (N = 3). Primers indicated in Supplementary Table 6. [file 40793_2021_381_MOESM2_ESM.pdf]

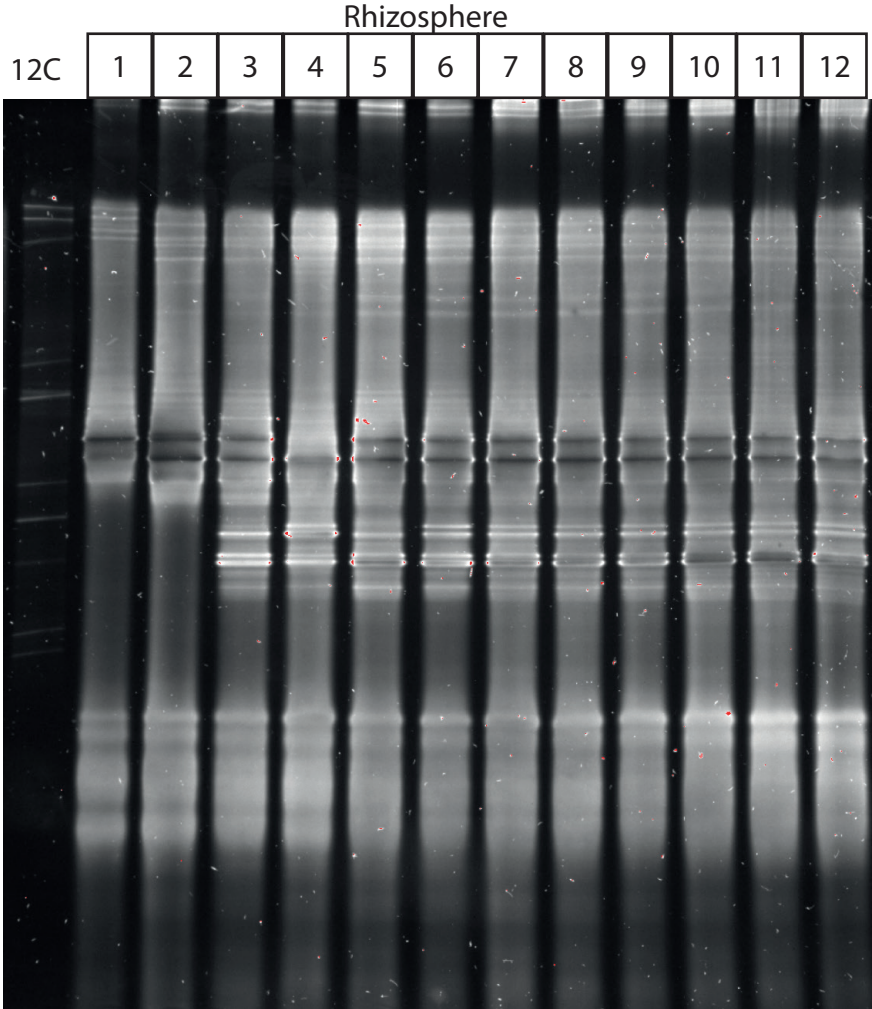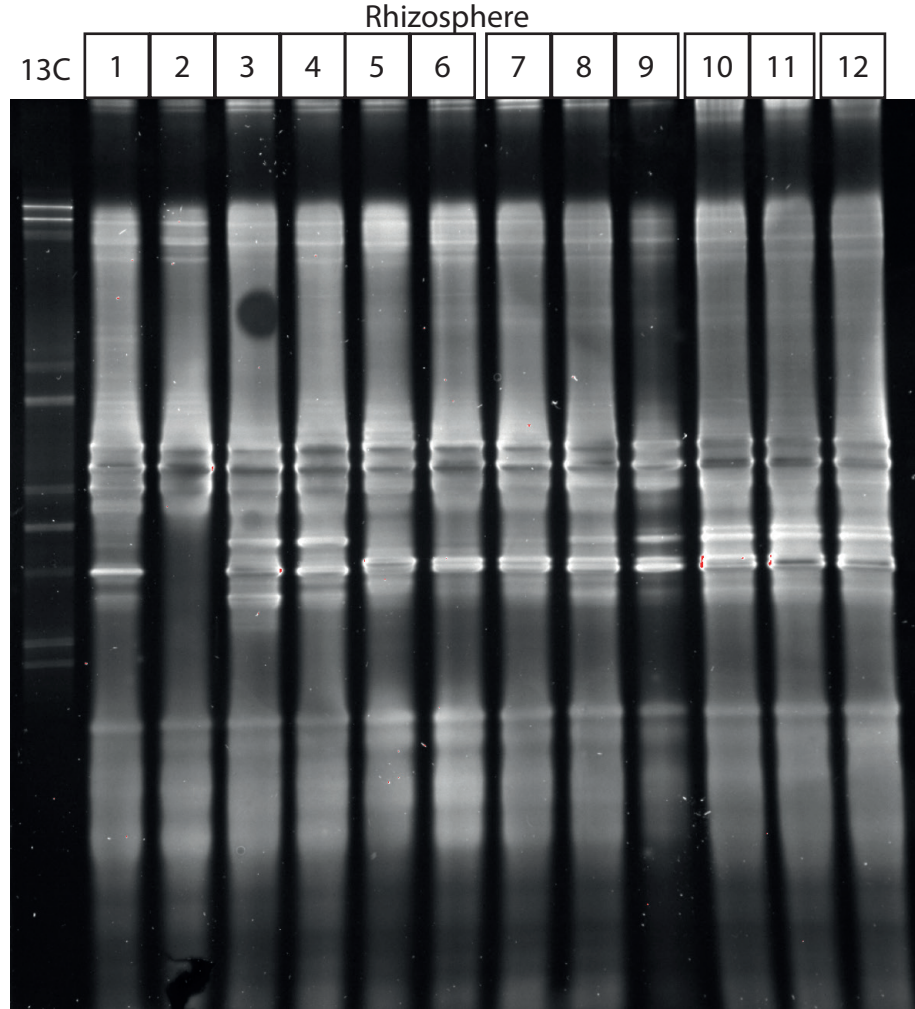

Supplement: Supplementary file 3 — Additional file 3: Supplementary Figure 3. Denaturing gel gradient electrophoresis (DGGE) showing archaeal 16S rRNA gene diversity across the 12 fractions generated for stable isotope probing for the rhizosphere associated with one 12C control (left) and 13C labelled (right) plant. Primers indicated in Supplementary Table 6. [file 40793_2021_381_MOESM3_ESM.pdf]

# A Rhizosphere

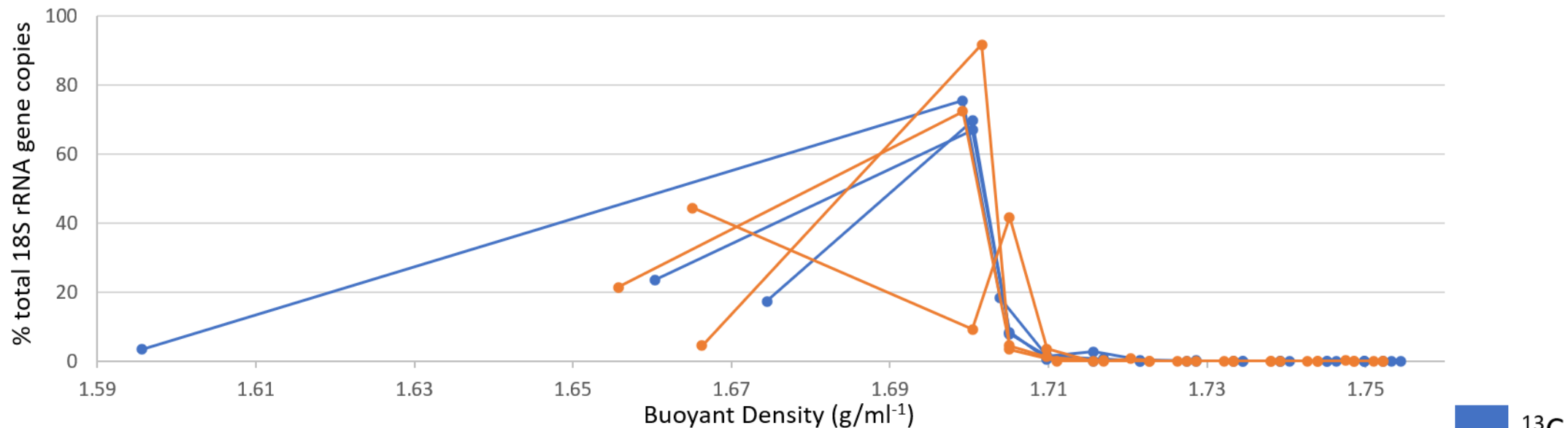

# B Endosphere

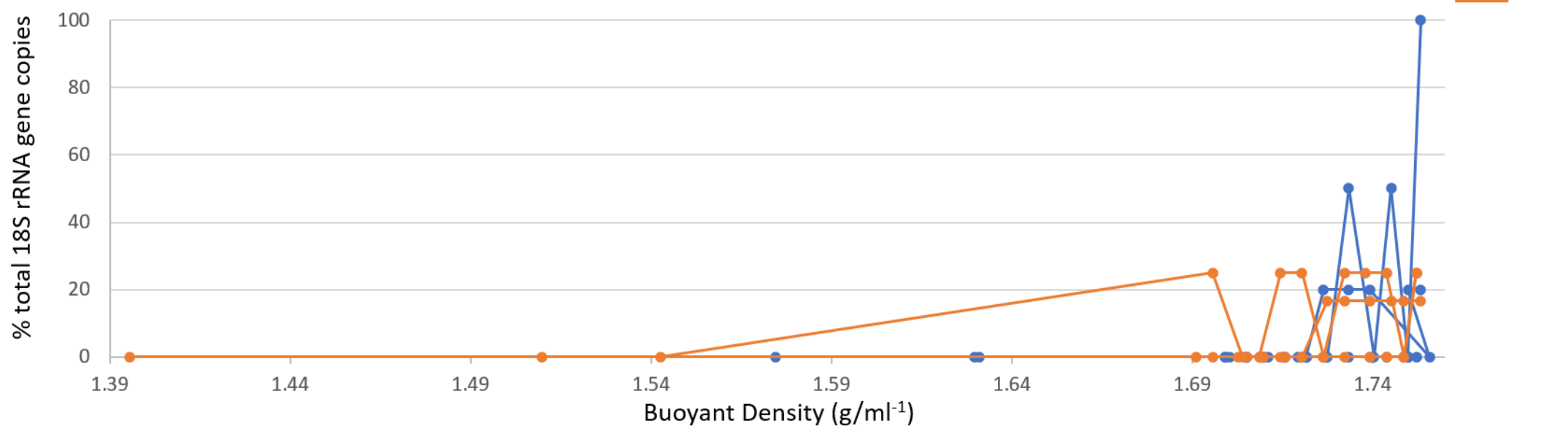

Supplement: Supplementary file 4 — Additional file 4: Supplementary Figure 4. Quantitative PCR against the fungal 18S rRNA gene to test for 13C labelling of the fungal community across fractions form the stable isotope probing. Graphs shows the percent of total 18S rRNA genes found within each of the 12 fractions for each plant (plotted as buoyant densities for that fraction in g / ml− 1) for 12C control and 13C labelled wheat plants from rhizosphere (A) and endosphere compartments (B) (N = 3). [file 40793_2021_381_MOESM4_ESM.pdf]

A

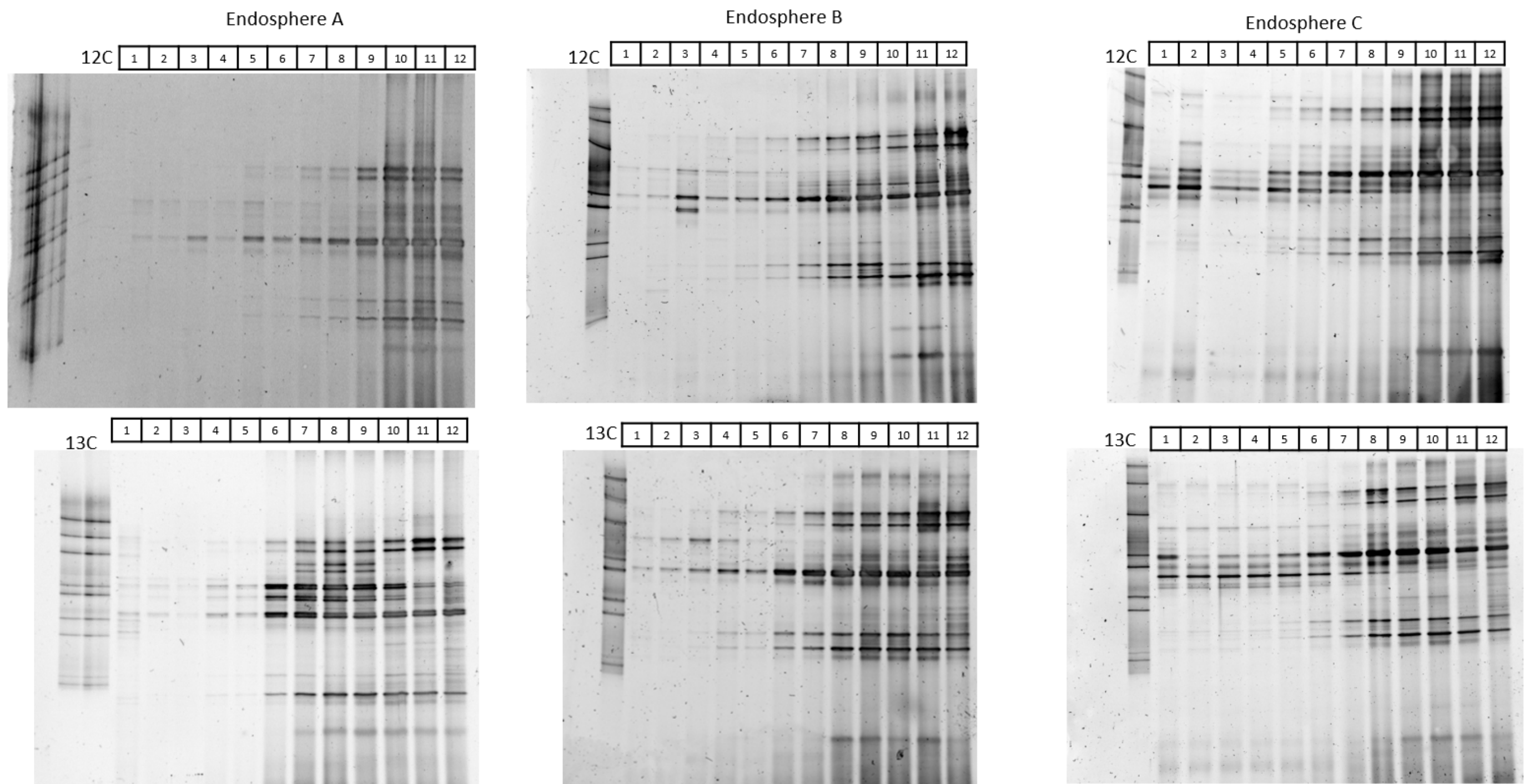

B

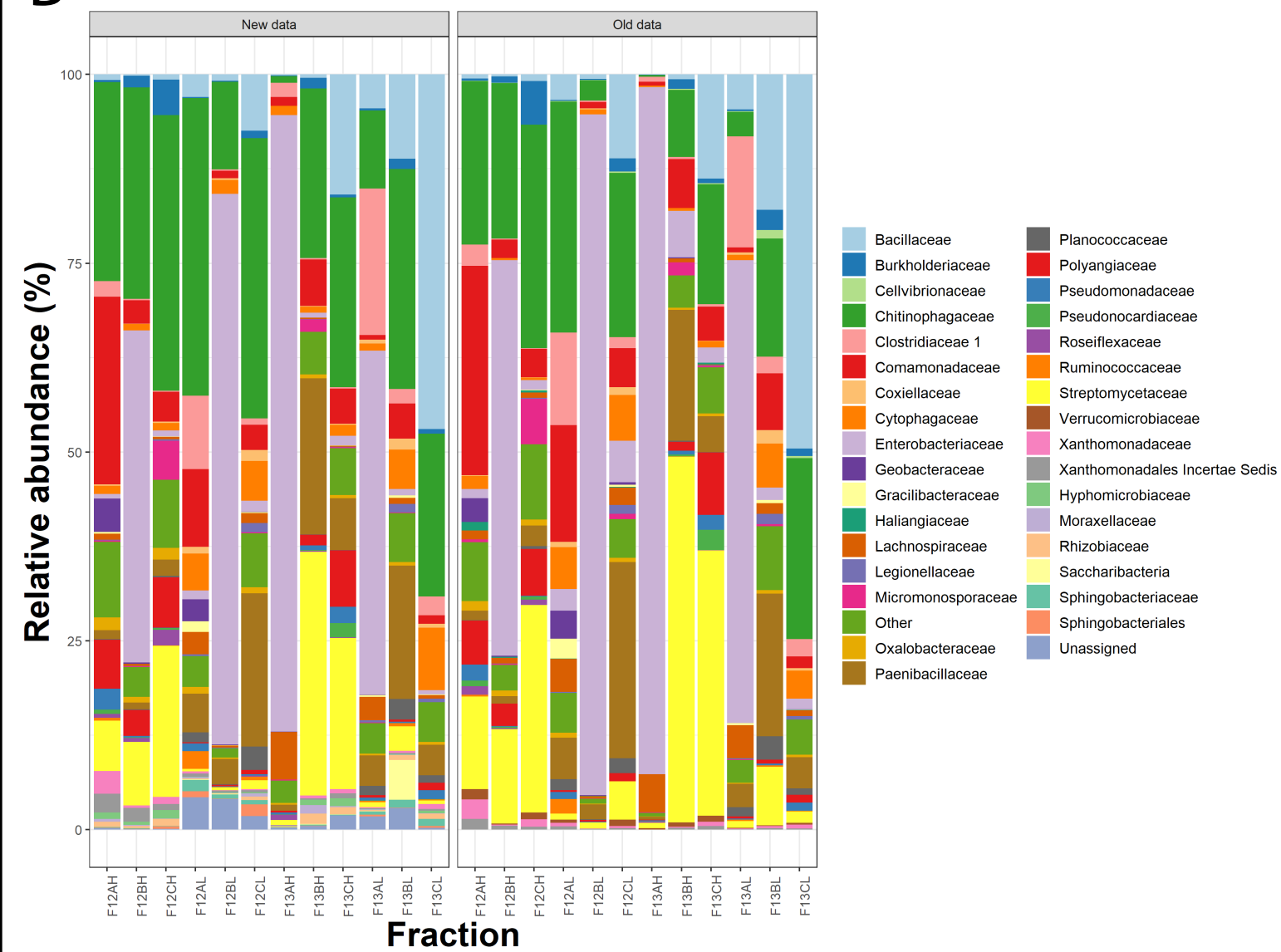

C

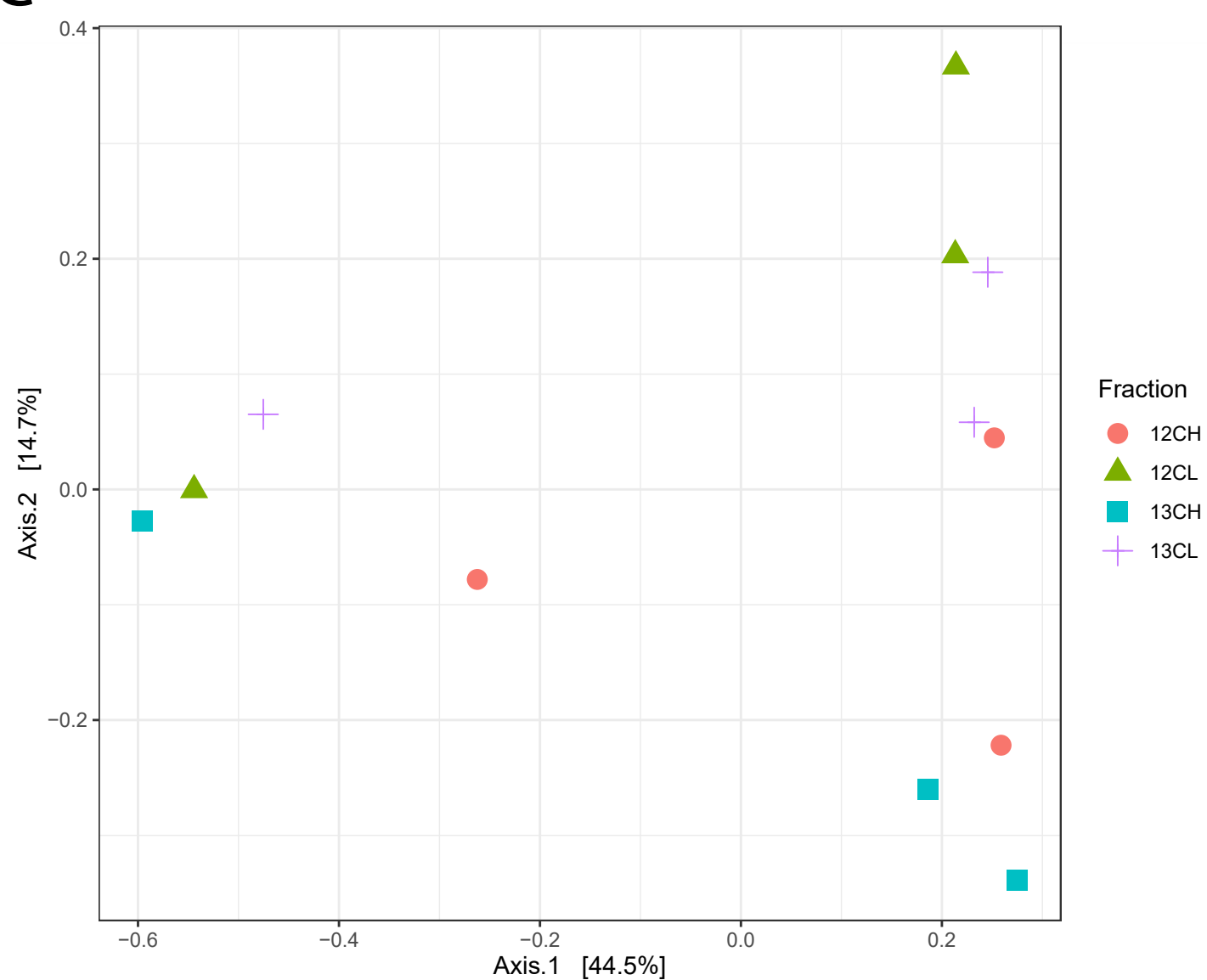

Supplement: Supplementary file 5 — Additional file 5: Supplementary Figure 5. Endosphere stable isotope probing data. A Denaturing gel gradient electrophoresis (DGGE) showing bacterial 16S rRNA gene diversity across the 12 fractions generated for stable isotope probing for the endosphere associated with three 12C control (top) and 13C labelled (bottom) plants. These gels show a shift in the bacterial community towards the heavy fraction of 13C labelled plants. B Bars show the relative abundance of each bacterial group within the pooled sequenced 12C heavy, 12C light, 13C heavy and 13C light fractions (N = 3), for two separate sequencing runs on the same samples (old & new). C Principle coordinates analysis (PCoA) on bray cutis dissimilarities for the endosphere 12C heavy (orange/circle), 12C light (green/triangle), 13C heavy (blue/square) and 13C light (purple/cross) fractions (N = 3). [file 40793_2021_381_MOESM5_ESM.pdf]

Bulk soil A

Bulk soil B

Bulk soil C

$^{13}\text{C}$

|   |   |   |   |   |   |   |   |   |    |    |    |
|---|---|---|---|---|---|---|---|---|----|----|----|
| 1 | 2 | 3 | 4 | 5 | 6 | 7 | 8 | 9 | 10 | 11 | 12 |
|---|---|---|---|---|---|---|---|---|----|----|----|

$^{13}\text{C}$

|   |   |   |   |   |   |   |   |   |    |    |    |
|---|---|---|---|---|---|---|---|---|----|----|----|
| 1 | 2 | 3 | 4 | 5 | 6 | 7 | 8 | 9 | 10 | 11 | 12 |
|---|---|---|---|---|---|---|---|---|----|----|----|

$^{13}\text{C}$

|   |   |   |   |   |   |   |   |   |    |    |    |
|---|---|---|---|---|---|---|---|---|----|----|----|
| 1 | 2 | 3 | 4 | 5 | 6 | 7 | 8 | 9 | 10 | 11 | 12 |
|---|---|---|---|---|---|---|---|---|----|----|----|

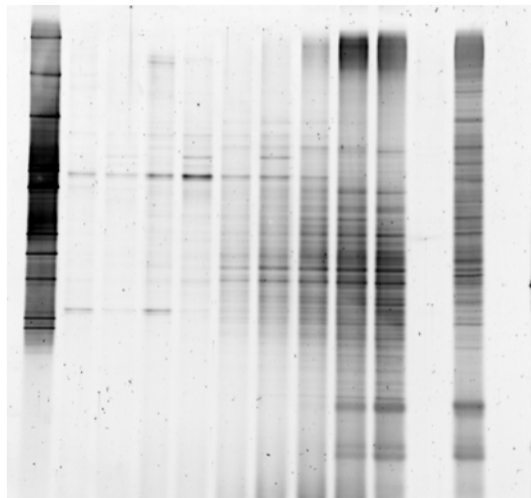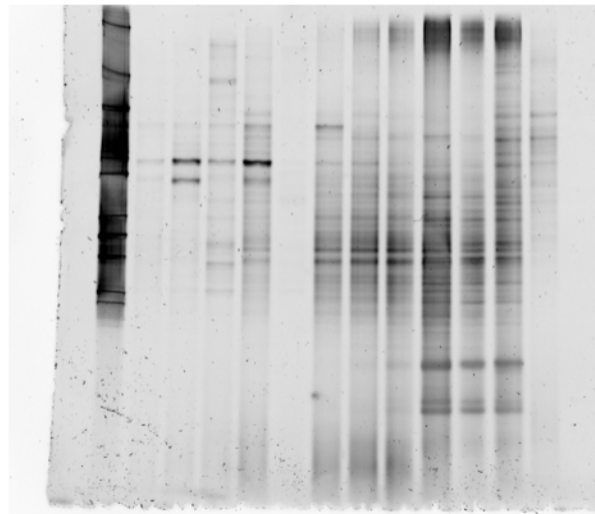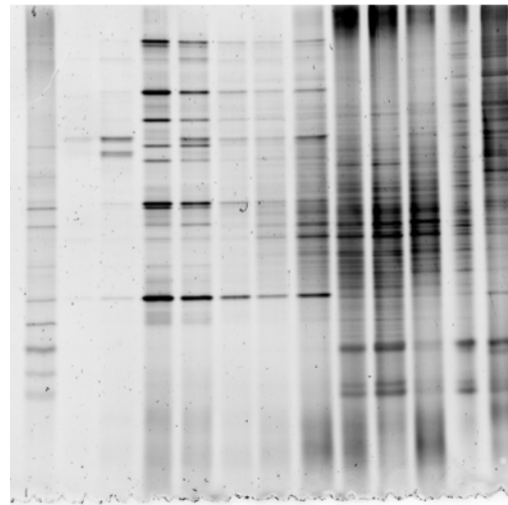

Supplement: Supplementary file 6 — Additional file 6: Supplementary Figure 6. Denaturing gel gradient electrophoresis (DGGE) showing bacterial 16S rRNA gene diversity across the 12 fractions generated for stable isotope probing for the 13C unplanted soil controls (N = 3). Primers indicated in Supplementary Table 6. [file 40793_2021_381_MOESM6_ESM.pdf]
