# Supplementary material for: Soil, senescence and exudate utilisation: characterisation of the Paragon var. spring bread wheat root microbiome
Source: Environ Microbiome. 2021 Jun 21;16:12. doi: 10.1186/s40793-021-00381-2 (PMC8215762; doi:10.1186/s40793-021-00381-2)
Supplement: Supplementary file 7 — Additional file 7: Supplementary Table 1. SIMPER outputs. Supplementary Table 2. Permanova results. Supplementary Table 3. Soil chemical properties. Supplementary Table 4. SIP fractions used for sequencing. Supplementary Table 5. DESeq2 outputs to identify root exudate utilisers in the rhizosphere. Supplementary Table 6. Primers. Supplementary Table 7. PCR Conditions for metabarcoding, qPCR & DGGE PCRs. Supplementary Table 8. Qiime 2 Dada2 settings. Supplementary Table 9. Qiime2 taxonomy-based filtering stats. Supplementary Table 10. DESeq2 output identifying CO2-fixing autotrophs from unplanted soil. Supplementary Table 11. DESeq2 outputs stem elongation field grown- Significantly differentially abundant bacterial taxa. Supplementary Table 12. DESeq2 outputs (all pot grown and stem elongation)- Significantly differentially abundant bacteria. Supplementary Table 13. DESeq2 outputs senescent plants- Significantly differentially abundant taxa. Supplementary Table 14. DESeq2 outputs stem elongation/senescent plants- Significantly differentially abundant bacterial taxa. Supplementary Table 15. DESeq2 outputs Significantly differentially abundant fungal taxa 01. Supplementary Table 16. DESeq2 outputs Significantly differentially abundant fungal taxa 02. [file 40793_2021_381_MOESM7_ESM.docx]

| Supplementary Table 1. SIMPER outputs | | |  |  |  |
| --- | --- | --- | --- | --- | --- |
| **Pot grown wheat, all soil types** | | | | | |
| **Comparison- Bulk soil-Endosphere** | | | **Bulk soil-Rhizosphere** | | |
| **OTU** | **Percent contribution** | **p-value** | **OTU** | **Percent contribution** | **p-value** |
| *Streptomycetaceae* | 14.6 | 0.0099 | *Burkholderiaceae* | 8.9 | 0.4851 |
| *Burkholderiaceae* | 10.1 | 0.0099 | *Rhizobiaceae* | 5.4 | 0.0099 |
| *Acidobacteria* Subgroup 6 | 3.6 | 0.0099 | *Acidobacteria* Subgroup 6 | 4 | 0.0198 |
| *Chitinophagaceae* | 3.5 | 0.0792 | *Bacillaceae* | 3.4 | 0.0594 |
| *Bacillaceae* | 2.8 | 0.0099 | *Micrococcaceae* | 3.1 | 0.0297 |
| *Xanthobacteraceae* | 2.6 | 0.0099 | *Chitinophagaceae* | 2.6 | 0.9108 |
| *Rhizobiaceae* | 2.4 | 0.6733 | *Xanthobacteraceae* | 2.5 | 0.7227 |
| Solirubrobacterales | 2 | 0.0099 | *Saccharimonadale* | 2.4 | 0.3168 |
| Saccharimonadales | 2 | 0.0792 | *Rubritaleaceae* | 2.3 | 0.0099 |
| *Methyloligellaceae* | 1.6 | 0.0099 | *Pseudomonadaceae* | 2.2 | 0.0099 |
| **Pot grown wheat, all soil types** | | | **Field-grown wheat** | | |
| **Comparison- Rhizosphere-Endosphere** | | | **Comparison- Bulk soil-Endosphere** | | |
| **OTU** | **Percent contribution** | **p-value** | **OTU** | **Percent contribution** | **p-value** |
| *Streptomycetaceae* | 16.7 | 0.0099 | *Streptomycetaceae* | 16.39547552 | 0.00990 |
| *Burkholderiaceae* | 7.2 | 0.7327 | *Burkholderiaceae* | 6.073676003 | 0.00990 |
| *Chitinophagaceae* | 3.8 | 0.3168 | *Firmicutes* | 5.602921016 | 0.00990 |
| *Rhizobiaceae* | 3.3 | 0.4752 | *Acidobacteria* Subgroup 6 | 5.547716313 | 0.00990 |
| *Micrococcaceae* | 3 | 0.0099 | *Sphingobacteriaceae* | 4.910748857 | 0.04950 |
| *Sphingobacteriaceae* | 2.4 | 0.1584 | *Bacillaceae* | 4.262550068 | 0.00990 |
| *Xanthobacteraceae* | 2.1 | 0.8416 | *Methyloligellaceae* | 2.413515939 | 0.00990 |
| Solirubrobacterales | 2.1 | 0.2277 | *Promicromonosporaceae* | 2.307694859 | 0.01980 |
| *Acidobacteria* Subgroup 6 | 2 | 1 | Solirubrobacterales | 2.038018274 | 0.00990 |
| **Field-grown wheat** | | | | | |
| **Bulk soil-Rhizosphere** | | | **Comparison- Rhizosphere-Endosphere** | | |
| **OTU** | **Percent contribution** | **p-value** | **OTU** | **Percent contribution** | **p-value** |
| *Sphingobacteriaceae* | 7.82061 | 0.10891 | *Streptomycetaceae* | 20.89116 | 0.01980 |
| *Burkholderiaceae* | 5.59509 | 0.77227 | *Firmicutes* | 7.499111 | 0.01980 |
| *Micrococcaceae* | 4.89642 | 0.10891 | *Micrococcaceae* | 4.78363 | 0.00990 |
| *Acidobacteria* Subgroup 6 | 4.74396 | 0.93069 | *Acidobacteria* Subgroup 6 | 4.240018 | 0.35643 |
| *Bacillaceae* | 4.08710 | 0.60396 | *Burkholderiaceae* | 4.209923 | 0.65346 |
| *Pseudomonadaceae* | 3.61463 | 0.00990 | *Sphingobacteriaceae* | 3.371828 | 0.98019 |
| *Rhizobiaceae* | 3.12084 | 0.00990 | *Bacillaceae* | 2.840192 | 0.68316 |
| *Methyloligellaceae* | 3.02557 | 0.17821 | *Pseudomonadaceae* | 2.130237 | 0.29703 |
| *Spirosomaceae* | 2.78842 | 0.17821 | *Promicromonosporaceae* | 2.044043 | 0.34653 |
| Solirubrobacterales | 2.42232 | 0.17821 | *Xanthobacteraceae* | 1.735366 | 0.02970 |

| Supplementary Table 2. PERMANOVA results | | | | | | |  | |  |
| --- | --- | --- | --- | --- | --- | --- | --- | --- | --- |
| **Stem elongation compared to pot agricultural** | | | | **Test for effect of root compartment on senescent communities** | | | | | |
| **Community = Archaea** | | | |  |  |  |  |  |  |
| **Compartment** | **Permutations** | **R^2^** | **p-value** | **Community** | | **Permutations** | **R^2^** | | **p-value** |
| Bulk Soil | 999 | 0.12 | 0.7 | Archaea | | 999 | 0.68 | | 0.01 |
| Rhizosphere | 999 | 0.12 | 0.7 | Bacteria | | 999 | 0.74 | | 0.005 |
| Endosphere | 999 | 0.34 | 0.2 | Fungi | | 999 | 0.73 | | 0.005 |
| **Community = Bacteria** | | | | **Test for effect of soil type on community composition** | | | | | |
| Bulk Soil | 999 | 0.3 | 0.1 | **Bulk Soil** | | | | | |
| Rhizosphere | 999 | 0.58 | 0.1 | Archaea | 999 | | | 0.94 | 0.003 |
| Endosphere | 999 | 0.53 | 0.1 | Bacteria | 999 | | | 0.87 | 0.001 |
| **Community = Fungi** | | | | Fungi | 999 | | | 0.81 | 0.004 |
| Bulk Soil | Fungi | 0.53 | 0.1 | **Rhizosphere** | | | | | |
| Rhizosphere | 999 | 0.39 | 0.1 | Archaea | 999 | | | 0.97 | 0.004 |
| Endosphere | 999 | 0.42 | 0.1 | Bacteria | 999 | | | 0.83 | 0.001 |
|  | | | | Fungi | 999 | | | 0.66 | 0.004 |
|  |  |  |  | **Endosphere** | | | | | |
|  |  |  |  | Archaea | 999 | | | 0.87 | 0.004 |
|  |  |  |  | Bacteria | 999 | | | 0.6 | 0.001 |

| Supplementary Table 3. Soil chemical properties | | |
| --- | --- | --- |
| **Parameter** | **Measurement** | **SAC Rating** |
| **Agricultural Soil, John Innes Centre Field Studies Site (sampled 18 04 2019)** | | |
| pH | 7.97 | n/a |
| Phosphorus (mg/kg) | 81.63 | High |
| Potassium (mg/kg) | 103 | Moderate |
| Magnesium (mg/kg) | 34.8 | Very Low |
| Nitrate (g/kg) | 73.92 | n/a |
| Ammonium (g/kg) | 6.97 | n/a |
| Organic matter (%) | 2.26 | n/a |
| **F2 Levington Compost** | | |
| pH | 4.98 | n/a |
| Phosphorus (mg/kg) | 880.5 | Excessively high |
| Potassium (mg/kg) | 2508 | Excessively high |
| Magnesium (mg/kg) | 6021 | Excessively high |
| Nitrate (g/kg) | 5809.49 | n/a |
| Ammonium (g/kg) | 192.18 | n/a |
| Organic matter (%) | 91.08 | n/a |

| Supplementary Table 4. SIP fractions used for sequencing | | | |
| --- | --- | --- | --- |
| **Origin of fractions** | **Fraction classification** | **Pooled fractions** | **fraction density range (g.ml^-1^)** |
| Endosphere A | 12H | 7,8,9 | 1.7086-1.7204 |
|  | 12L | 11,12 | 1.3956-1.6910 |
|  | 13H | 7,8,9 | 1.7098-1.7227 |
|  | 13L | 11,12 | 1.6004-1.6992 |
| Endosphere B | 12H | 7,8,9 | 1.7098-1.7204 |
|  | 12L | 11,12 | 1.5097-1.6957 |
|  | 13H | 7,8,9 | 1.7098-1.7216 |
|  | 13L | 11,12 | 1.5745-1.6992 |
| Endosphere C | 12H | 7,8,9 | 1.7086-1.7204 |
|  | 12L | 11,12 | 1.5427-1.6957 |
|  | 13H | 7,8,9 | 1.7110-1.7216 |
|  | 13L | 11,12 | 1.6310-1.7004 |
| Rhizosphere A | 12H | 7,8 | 1.7169-1.7227 |
|  | 12L | 10,11 | 1.6992-1.7051 |
|  | 13H | 7,8 | 1.7169-1.7216 |
|  | 13L | 10,11 | 1.6992-1.7039 |
| Rhizosphere B | 12H | 7,8 | 1.7157-1.7204 |
|  | 12L | 10,11 | 1.7004-1.7051 |
|  | 13H | 7,8 | 1.7157-1.7216 |
|  | 13L | 10,11 | 1.7004-1.7051 |
| Rhizosphere C | 12H | 7,8 | 1.7169-1.7227 |
|  | 12L | 10,11 | 1.7016-1.7051 |
|  | 13H | 7,8 | 1.7157-1.7216 |
|  | 13L | 10,11 | 1.7004-1.7051 |
| Unplanted A | 13H | 8,9 | 1.7086-1.7145 |
|  | 13L | 11 | 1.6992 |
| Unplanted B | 13H | 8 | 1.7145 |
|  | 13L | 10,11 | 1.7004-1.7039 |
| Unplanted C | 13H | 8,9 | 1.7098-1.7157 |
|  | 13L | 11,12 | 1.6745-1.7004 |

| Supplementary Table 5. DESeq2 outputs to identify root exudate utilisers in the rhizosphere | | | | |
| --- | --- | --- | --- | --- |
| **^12^C heavy compared to ^13^C heavy** | | | | |
| **OTU** | **baseMean** | **log2FoldChange** | **lfsSE *** | **Padj** |
| *Enterobacteriaceae* | 1525.585247 | 9.225478608 | 1.330868073 | 8.30E-11 |
| *Paenibacillaceae* | 542.7147998 | 6.131222282 | 0.71096459 | 1.62E-16 |
| *Verrucomicrobiaceae* | 1540.476332 | 5.525652719 | 0.38986191 | 1.34E-43 |
| *Pseudomonadaceae* | 747.4943245 | 5.296358431 | 1.327440714 | 0.000347898 |
| *Oxalobacteraceae* | 1121.798139 | 5.093564169 | 0.466396414 | 3.05E-26 |
| *Cellvibrionaceae* | 143.5596665 | 4.563865623 | 0.692665528 | 6.33E-10 |
| *Comamonadaceae* | 1965.095913 | 4.553967836 | 0.399631593 | 2.20E-28 |
| *Fibrobacteraceae* | 308.4266565 | 4.250982079 | 0.691291828 | 9.73E-09 |
| *Rhizobiaceae* | 200.6141532 | 3.586599358 | 0.543771655 | 6.33E-10 |
| *Cytophagaceae* | 333.3918811 | 3.485162531 | 0.695142566 | 4.45E-06 |
| *Micrococcaceae* | 1002.919707 | 3.188857943 | 0.72279587 | 6.41E-05 |
| *Microbacteriaceae* | 182.5423665 | 2.316546635 | 0.708211293 | 0.004465608 |
| *Xanthomonadaceae* | 191.2474009 | 2.210211572 | 0.38114642 | 6.68E-08 |
| *Intrasporangiaceae* | 196.5957779 | 1.679541697 | 0.522170126 | 0.005191223 |
| *Polyangiaceae* | 174.8113772 | 1.441127742 | 0.521692567 | 0.016393349 |
| **^13^C light compared to ^13^C heavy** | | | | |
| **OTU** | **baseMean** | **log2FoldChange** | **lfsSE *** | **Padj** |
| *Enterobacteriaceae* | 1193.557819 | 5.663078218 | 1.200852813 | 1.47E-05 |
| *Paenibacillaceae* | 474.7391477 | 3.450277252 | 0.717541055 | 1.06E-05 |
| *Verrucomicrobiaceae* | 1240.232555 | 4.817514423 | 0.470756764 | 4.58E-23 |
| *Pseudomonadaceae* | 640.932411 | 2.82797024 | 1.152315253 | 0.034597109 |
| *Oxalobacteraceae* | 882.3908862 | 6.866216698 | 0.630972123 | 6.88E-26 |
| *Cellvibrionaceae* | 122.6921288 | 3.359934177 | 0.602489726 | 3.00E-07 |
| *Comamonadaceae* | 1557.064356 | 4.810895677 | 0.375167998 | 1.19E-35 |
| *Fibrobacteraceae* | 247.2869102 | 3.558583564 | 0.645095913 | 3.77E-07 |
| *Rhizobiaceae* | 194.8884915 | 1.676668472 | 0.519657919 | 0.004548681 |
| *Cytophagaceae* | 283.9658689 | 2.520107112 | 0.595381826 | 0.000113113 |
| *Micrococcaceae* | 918.575397 | 1.673919306 | 0.649494375 | 0.025268353 |
| *Microbacteriaceae* | 144.683441 | 2.15700887 | 0.693944173 | 0.006585131 |
| *Xanthomonadaceae* | 167.3075238 | 1.56520526 | 0.53711052 | 0.011040717 |
| *Intrasporangiaceae* | 158.6061631 | 1.50689272 | 0.517691543 | 0.011040717 |
| *Polyangiaceae* | 119.7725455 | 2.424270358 | 0.530116698 | 2.62E-05 |
| *lfsSE= log2 Fold Change Standard Error | | | | |

| Supplementary Table 6. Primers | | | | |
| --- | --- | --- | --- | --- |
| **Primer** | **Target** | **Uses** | **Sequence** | **Reference** |
| PRK341F-GC | Bacterial 16S | DGGE PCR Amplification | CGCCCGCCGCGCGCGGCGGGCGGGGCGGGGGCACGGGGGGCCTACGGGAGGCAGCAG | (Muyzer *et al.*, 1993) [1] |
| 518R | Bacterial 16S | DGGE PCR Amplification | ATTACCGCGGCTGCTGG | (Muyzer *et al.*, 1993) [1] |
| A771F-GC | Archaeal 16S | DGGE PCR Amplification | CGCCCGCCGCGCGCGGCGGG  CGGGGCGGGGGCACGGGGGG  ACGGTGAGGGATGAAAGCT | (Ochsenreiter *et al.*, 2003) [2] |
| A957R | Archaeal 16S | DGGE PCR Amplification, qPCR | CGGCGTTGACTCCAATTG | (Ochsenreiter *et al.*, 2003) [2] |
| A109F | Archaeal 16S | PCR amplification, qPCR standard amplification | ACKGCTCAGTAACACGT | (Großkopf *et al.*, 1998) [3] |
| A1000R | Archaeal 16S | PCR amplification, qPCR standard amplification | GGCCATGCACYWCYTCTC | (Gantner *et al.*, 2011) [4] |
| PRK341F | Bacterial 16S | PCR amplification/ Sequencing, qPCR standard amplification | CCTACGGGRBGCASCAG | (Yu *et al*., 2005) [5] |
| MPRK806R | Bacterial 16S | PCR amplification/ Sequencing, qPCR standard amplification | GGACTACNNGGGTATCTAAT | (Yu *et al.*, 2005) [5] |
| fITS7F | Fungal ITS2 | PCR Amplification/ sequencing | GTGARTCATCGAATCTTTG | (Ihrmark *et al.*, 2012) [6] |
| ITS4R_2 | Fungal ITS2 | PCR Amplification/ sequencing | TCCTCCGCTTATTGATATGC | (White *et al*., 1990) [7] |
| A771F | Archaeal 16S | qPCR | ACGGTGAGGGATGAAAGCT | (Ochsenreiter *et al.*, 2003) [2] |
| FR1Fw | Fungal 18S | qPCR | AICCATTCAATCGGTAIT | (Vainio *et al.*, 2000) [8] |
| FF390Rev | Fungal 18S | qPCR | CGATAACGAACGAGACCT | (Vainio *et al.*, 2000) [8] |
| Com1F | Bacterial 16S | qPCR | CAGCAGCCGCGGTAATAC | (Fredriksson *et al*., 2013) [9] |
| 769R | Bacterial 16S | qPCR | ATCCTGTTTGMTMCCCVCRC | (Rastogi *et al*., 2010) [10] |
| F18SS03-F | Fungal 18S | qPCR standard amplification | AGATCCTGAGGCCTCACTA | This Study |
| F18SS03-R | Fungal 18S | qPCR standard amplification | GCCGTTCTTAGTTGGTGGAG | This Study |
| A0349F | Archaeal 16S | Sequencing | GYGCASCAGKCGMGAAW | (Takai *et al.*, 2000) [11] |
| A0519R | Archaeal 16S | Sequencing | TTACCGCGGCKGCTG | (Takai *et al.*, 2000) [11] |
| CrenamoA23f | Archaeal *amoA* | DGGE | ATGGTCTGGCTWAGACG | (Tourna *et al*., 2008) [12] |
| CrenamoA616r | Archaeal *amoA* | DGGE | GCCATCCATCTGTATGTCCA | (Tourna *et al*., 2008) [12] |

| Supplementary Table 7. PCR Conditions for metabarcoding, qPCR & DGGE PCRs | | |
| --- | --- | --- |
| **PCR Component** | | **Volume (µl)** |
| 2x PCRBio BioMix™ red, containing BIOTAQ™ DNA Polymerase or 2x PCRBio Ultra mix, containing Ultra DNA Polymerase | | 10 |
| Forward or reverse primer (10mM stock) | | 1 |
| Template DNA | | 2 (DNA extract)  1 (Round 1 product for nested PCR) |
| Sterile dH_2_O | | Up to 20µl |
| **qPCR mix** | | |
| 2x SYBR Green Luna® Universal qPCR Master Mix | | 10µl |
| Forward or reverse primer (10mM stock) | | 0.5µl |
| DNA template (20ng/µl stock) | | 5µl |
| Sterile MilliQ dH_2_O | | Up to 20µl |
| **Thermocycler programs** | | |
| A0109F/A1000R  Archaeal 16S rRNA gene  For sequencing & round one of DGGE | 1. 95°C for 1 minute 2. 35x cycles of 95°C for 30 seconds, 59°C for 30 seconds, 72°C for 45 seconds 3. 72°C for 1 minute | |
| A771F-GC/A957R  Archaeal 16S rRNA gene  For round two of DGGE | 1. 95°C for 1 minute 2. 35 cycles of 94°C for 30 seconds, 55°C for 30 seconds, 72°C for 1 minute 3. 72°C for 10 minutes | |
| PRK341F/MPRK806R or fITS7F/ITS4R  Bacterial 16S rRNA gene or Fungal ITS2 region  For sequencing | 1. 95°C for 1 minute 2. 30 cycles of 95°C for 15 seconds, 55°C for 15 seconds, 72°C for 15 seconds 3. 72°C for 10 minutes | |
| A771F/A957R, FR1Fw/FF390Rev or Com1F/769R qPCR assays | 1. 95°C for 10 minutes 2. 35 cycles of 95°C for 15 seconds and 60°C annealing/extension/read step for 30 seconds | |

Supplementary Table 8. Qiime 2 Dada2 settings

| **Amplicon** | **p-trim-left-f** | **p-trim-left-r** | **p-trunc-len-f/r** |
| --- | --- | --- | --- |
| A0349F/A0519R Archaeal 16S | 17 | 15 | 120 |
| PRK341F/ MPRK806R Bacterial 16S | 17 | 20 | 230 |
| fITS7F/ ITS4R Fungal ITS2 | 19 | 20 | 195 |

| Supplementary Table 9. Qiime2 taxonomy-based filtering stats | | | | | | |
| --- | --- | --- | --- | --- | --- | --- |
| **Experiment** | **Amplicon** | **Sample** | **Total No. trimmed quality filtered reads** | **No. reads removed by taxonomic filtering** | **No. reads remaining** | **% reads discarded** |
| Metabarcoding | A0349F/A0519R Archaeal 16S | BS51.A | 68773 | 475 | 68298 | 0.690678 |
|  |  | BS52.A | 63866 | 625 | 63241 | 0.978611 |
|  |  | BS53.A | 74447 | 575 | 73872 | 0.772362 |
|  |  | BSA1.A | 55327 | 867 | 54460 | 1.567047 |
|  |  | BSA2.A | 54695 | 195 | 54500 | 0.356523 |
|  |  | BSA3.A | 49970 | 390 | 49580 | 0.780468 |
|  |  | BSF1.A | 78012 | 1695 | 76317 | 2.172743 |
|  |  | BSF2.A | 75560 | 1892 | 73668 | 2.50397 |
|  |  | BSF3.A | 78741 | 1574 | 77167 | 1.998959 |
|  |  | BSL1.A | 207703 | 11458 | 196245 | 5.516531 |
|  |  | BSL2.A | 196347 | 8785 | 187562 | 4.474222 |
|  |  | BSL3.A | 133107 | 4169 | 128938 | 3.132067 |
|  |  | E51.A | 93810 | 119 | 93691 | 0.126852 |
|  |  | E52.A | 92550 | 711 | 91839 | 0.768233 |
|  |  | E53.A | 109472 | 330 | 109142 | 0.301447 |
|  |  | EA1.A | 79035 | 54 | 78981 | 0.068324 |
|  |  | EA2.A | 72672 | 6124 | 66548 | 8.426904 |
|  |  | EA3.A | 58574 | 34 | 58540 | 0.058046 |
|  |  | EF1.A | 78178 | 2079 | 76099 | 2.659316 |
|  |  | EF2.A | 59310 | 442 | 58868 | 0.745237 |
|  |  | EF3.A | 57468 | 856 | 56612 | 1.489525 |
|  |  | EL1.A | 126246 | 159 | 126087 | 0.125945 |
|  |  | EL2.A | 133715 | 200 | 133515 | 0.149572 |
|  |  | RZ51.A | 69659 | 2320 | 67339 | 3.33051 |
|  |  | RZ52.A | 75587 | 1549 | 74038 | 2.049294 |
|  |  | RZ53.A | 68546 | 1174 | 67372 | 1.712718 |
|  |  | RZA1.A | 58037 | 1202 | 56835 | 2.071093 |
|  |  | RZA2.A | 90071 | 1176 | 88895 | 1.305637 |
|  |  | RZA3.A | 68877 | 1513 | 67364 | 2.196669 |
|  |  | RZF1.A | 37285 | 304 | 36981 | 0.815341 |
|  |  | RZF2.A | 48495 | 321 | 48174 | 0.661924 |
|  |  | RZF3.A | 56662 | 657 | 56005 | 1.159507 |
|  |  | RZL1.A | 125065 | 3873 | 121192 | 3.09679 |
|  |  | RZL2.A | 125751 | 3886 | 121865 | 3.090234 |
|  |  | RZL3.A | 123964 | 2723 | 121241 | 2.196605 |
|  |  | EFS1.A | 239780 | 2790 | 236990 | 1.1 |
|  |  | EFS2.A | 247035 | 643 | 246392 | 0.260287 |
|  |  | EFS3.A | 199509 | 1400 | 198109 | 0.701723 |
|  |  | RZFS1.A | 247152 | 881 | 246271 | 0.356461 |
|  |  | RZFS2.A | 239414 | 935 | 238479 | 0.390537 |
|  |  | RZFS3.A | 241137 | 280 | 240857 | 0.116117 |
|  |  | BSFS1.A | 254643 | 226 | 254417 | 0.088752 |
|  |  | BSFS2.A | 304279 | 289 | 303990 | 0.949786 |
|  |  | BSFS3.A | 478179 | 808 | 477371 | 0.168974 |
|  | PRK341F/ MPRK806R Bacterial 16S | B.BS51 | 22970 | 320 | 22650 | 1.393121 |
|  |  | B.BS52 | 12809 | 510 | 12299 | 3.981575 |
|  |  | B.BS53 | 22638 | 396 | 22242 | 1.749271 |
|  |  | B.BSA1 | 13386 | 455 | 12931 | 3.399074 |
|  |  | B.BSA2 | 18260 | 778 | 17482 | 4.260679 |
|  |  | B.BSA3 | 17575 | 977 | 16598 | 5.559033 |
|  |  | B.BSL1 | 32363 | 3322 | 29041 | 10.26481 |
|  |  | B.BSL2 | 28475 | 3580 | 24895 | 12.57243 |
|  |  | B.BSL3 | 28042 | 3478 | 24564 | 12.40282 |
|  |  | B.E51 | 51847 | 50333 | 1514 | 97.07987 |
|  |  | B.E52 | 46632 | 46145 | 487 | 98.95565 |
|  |  | B.E53 | 49496 | 46634 | 2862 | 94.21771 |
|  |  | B.EA1 | 46470 | 42828 | 3642 | 92.16269 |
|  |  | B.EA2 | 49475 | 47264 | 2211 | 95.53108 |
|  |  | B.EA3 | 39328 | 32351 | 6977 | 82.25946 |
|  |  | B.EL1 | 40368 | 39732 | 636 | 98.42449 |
|  |  | B.EL2 | 46651 | 46125 | 526 | 98.87248 |
|  |  | B.EL3 | 48154 | 46843 | 1311 | 97.27748 |
|  |  | B.RZ51 | 17754 | 976 | 16778 | 5.497353 |
|  |  | B.RZ52 | 18840 | 983 | 17857 | 5.217622 |
|  |  | B.RZ53 | 17688 | 782 | 16906 | 4.421076 |
|  |  | B.RZA1 | 22737 | 687 | 22050 | 3.021507 |
|  |  | B.RZA2 | 23577 | 813 | 22764 | 3.448276 |
|  |  | B.RZA3 | 20817 | 647 | 20170 | 3.108037 |
|  |  | B.RZL1 | 21777 | 632 | 21145 | 2.902144 |
|  |  | B.RZL2 | 26411 | 1015 | 25396 | 3.843096 |
|  |  | B.RZL3 | 27594 | 1896 | 25698 | 6.871059 |
|  |  | B.BSF1 | 17020 | 1101 | 15919 | 6.46886 |
|  |  | B.BSF2 | 15340 | 568 | 14772 | 3.702738 |
|  |  | B.BSF3 | 19415 | 414 | 19001 | 2.132372 |
|  |  | B.EF1 | 41256 | 24671 | 16585 | 59.79979 |
|  |  | B.EF2 | 42388 | 27888 | 14500 | 65.79221 |
|  |  | B.EF3 | 44801 | 27308 | 17493 | 60.954 |
|  |  | B.RZF1 | 21359 | 1201 | 20158 | 5.622922 |
|  |  | B.RZF2 | 24495 | 472 | 24023 | 1.926924 |
|  |  | B.RZF3 | 19582 | 760 | 18822 | 3.881115 |
|  |  | B.EFS1 | 53449 | 138 | 53311 | 0.25819 |
|  |  | B.EFS2 | 60192 | 350 | 59842 | 0.581473 |
|  |  | B.EFS3 | 54148 | 755 | 53393 | 1.394327 |
|  |  | B.RZFS1 | 47685 | 367 | 47318 | 0.769341 |
|  |  | B.RZFS2 | 46770 | 2122 | 44648 | 4.537096 |
|  |  | B.RZFS3 | 48877 | 844 | 48033 | 1.726784 |
|  |  | B.BSFS1 | 43114 | 7244 | 35870 | 16.80197 |
|  |  | B.BSFS2 | 42848 | 4569 | 38279 | 10.66327 |
|  |  | B.BSFS3 | 48794 | 6410 | 42384 | 13.13686 |
|  | fITS7F/ ITS4R Fungal ITS2 | F.BS51 | 41729 | n/a | n/a | n/a |
|  |  | F.BS52 | 45565 | n/a | n/a | n/a |
|  |  | F.BS53 | 46115 | n/a | n/a | n/a |
|  |  | F.BSA1 | 75779 | n/a | n/a | n/a |
|  |  | F.BSA2 | 100787 | n/a | n/a | n/a |
|  |  | F.BSA3 | 94052 | n/a | n/a | n/a |
|  |  | F.BSF1 | 129866 | n/a | n/a | n/a |
|  |  | F.BSF2 | 100424 | n/a | n/a | n/a |
|  |  | F.BSF3 | 81109 | n/a | n/a | n/a |
|  |  | F.BSL1 | 98179 | n/a | n/a | n/a |
|  |  | F.BSL2 | 131559 | n/a | n/a | n/a |
|  |  | F.BSL3 | 120348 | n/a | n/a | n/a |
|  |  | F.E51 | 101772 | n/a | n/a | n/a |
|  |  | F.E52 | 114929 | n/a | n/a | n/a |
|  |  | F.E53 | 44992 | n/a | n/a | n/a |
|  |  | F.EA1 | 75592 | n/a | n/a | n/a |
|  |  | F.EA2 | 65668 | n/a | n/a | n/a |
|  |  | F.EA3 | 89945 | n/a | n/a | n/a |
|  |  | F.EF1 | 89057 | n/a | n/a | n/a |
|  |  | F.EF2 | 69637 | n/a | n/a | n/a |
|  |  | F.EF3 | 102551 | n/a | n/a | n/a |
|  |  | F.RZ51 | 84654 | n/a | n/a | n/a |
|  |  | F.RZ52 | 37011 | n/a | n/a | n/a |
|  |  | F.RZ53 | 31117 | n/a | n/a | n/a |
|  |  | F.RZA1 | 102095 | n/a | n/a | n/a |
|  |  | F.RZA2 | 71665 | n/a | n/a | n/a |
|  |  | F.RZA3 | 84015 | n/a | n/a | n/a |
|  |  | F.RZF1 | 51408 | n/a | n/a | n/a |
|  |  | F.RZF2 | 99541 | n/a | n/a | n/a |
|  |  | F.RZF3 | 45291 | n/a | n/a | n/a |
|  |  | F.RZL1 | 110595 | n/a | n/a | n/a |
|  |  | F.RZL2 | 97230 | n/a | n/a | n/a |
|  |  | F.RZL3 | 81901 | n/a | n/a | n/a |
|  |  | F.EFS1 | 129650 | n/a | n/a | n/a |
|  |  | F.EFS2 | 70055 | n/a | n/a | n/a |
|  |  | F.EFS3 | 76512 | n/a | n/a | n/a |
|  |  | F.RZFS1 | 56010 | n/a | n/a | n/a |
|  |  | F.RZFS2 | 64946 | n/a | n/a | n/a |
|  |  | F.RZFS3 | 36457 | n/a | n/a | n/a |
|  |  | F.BSFS1 | 104076 | n/a | n/a | n/a |
|  |  | F.BSFS2 | 71894 | n/a | n/a | n/a |
|  |  | F.BSFS3 | 80601 | n/a | n/a | n/a |
| Endosphere SIP sequencing (1st run) | PRK341F/ MPRK806R Bacterial 16S | 12CHa | 93805 | 43076 | 50729 | 45.92079 |
|  |  | 12CLa | 116684 | 89134 | 27550 | 76.38922 |
|  |  | 13CHa | 108182 | 29914 | 78268 | 27.65155 |
|  |  | 13CLa | 97947 | 48972 | 48975 | 49.99847 |
|  |  | 12CHb | 108089 | 43142 | 64947 | 39.9134 |
|  |  | 12CLb | 105021 | 50010 | 55011 | 47.61905 |
|  |  | 13CHb | 95683 | 73700 | 21983 | 77.02518 |
|  |  | 13CLb | 71120 | 52215 | 18905 | 73.41817 |
|  |  | 12CHb | 100291 | 56735 | 43556 | 56.57038 |
|  |  | 12CLb | 92092 | 84369 | 7723 | 91.61382 |
|  |  | 13CHb | 83840 | 46040 | 37800 | 54.91412 |
|  |  | 13CLb | 85336 | 45571 | 39765 | 53.40185 |
| Rhizosphere and Bulk soil SIP sequencing |  | 12CHa | 13421 | 885 | 12536 | 6.594144 |
|  |  | 12CLa | 12970 | 798 | 12172 | 6.15266 |
|  |  | 13CHa | 15960 | 336 | 15624 | 2.105263 |
|  |  | 13CLa | 12166 | 142 | 12024 | 1.167187 |
|  |  | 12CHb | 22378 | 3195 | 19183 | 14.27742 |
|  |  | 12CLb | 14140 | 986 | 13154 | 6.973126 |
|  |  | 13CHb | 14082 | 321 | 13761 | 2.279506 |
|  |  | 13CLb | 10732 | 286 | 10446 | 2.664927 |
|  |  | 12CHc | 15785 | 316 | 15469 | 2.001901 |
|  |  | 12CLc | 8832 | 380 | 8452 | 4.302536 |
|  |  | 13CHc | 12383 | 172 | 12211 | 1.389001 |
|  |  | 13CLc | 10651 | 146 | 10505 | 1.370763 |
|  |  | 13CHa | 19395 | 4230 | 15165 | 21.80974 |
|  |  | 13CLa | 14826 | 1932 | 12894 | 13.03116 |
|  |  | 13CHb | 19529 | 1409 | 18120 | 7.214911 |
|  |  | 13CLb | 17501 | 1740 | 15761 | 9.942289 |
|  |  | 13CHc | 15543 | 2230 | 13313 | 14.34729 |
|  |  | 13CLc | 18138 | 2585 | 15553 | 14.25185 |
| Endosphere SIP sequencing (2nd run) |  | 12CHa | 129499 | 76038 | 53461 | 41.28294 |
|  |  | 12CLa | 112494 | 35149 | 77345 | 68.75478 |
|  |  | 12CHb | 129363 | 68584 | 60779 | 46.9833 |
|  |  | 12CLb | 128990 | 61089 | 67901 | 52.64051 |
|  |  | 12CHc | 111366 | 56655 | 54711 | 49.1272 |
|  |  | 12CLc | 106026 | 27714 | 78312 | 73.86113 |
|  |  | 13CHa | 145802 | 94298 | 51504 | 35.32462 |
|  |  | 13CLa | 102901 | 53396 | 49505 | 48.10935 |
|  |  | 13CHb | 104884 | 40712 | 64172 | 61.18378 |
|  |  | 13CLb | 92606 | 31200 | 61406 | 66.30888 |
|  |  | 13CHc | 124416 | 57972 | 66444 | 53.40471 |
|  |  | 13CLc | 109262 | 53343 | 55919 | 51.17882 |
|  |  | **Key**: BS = Bulk Soil, RZ = Rhizosphere, E = Endosphere  5 = 50:50 Mix, A = Agricltural Pot, F = Stem Elongation Field, L = Levington F2 Compost, FS = Senescent Field  1/2/3 = sample replicate, A./B./F./ = Archaeal/Bacterial/Fungal amplicon, 12CL – 12C light, 12CH – 12C heavy, 13CL – 13C light, 13CH – 13C heavy, a/b/c – sample replicate. | | | | |

| Supplementary Table 10. DESeq2 output identifying CO_2_-fixing autotrophs from unplanted soil | | | | |
| --- | --- | --- | --- | --- |
| **OTU** | **baseMean** | **log2FoldChange** | **lfsSE *** | **padj** |
| *Gaiellaceae* | 523.2173795 | 2.423212929 | 0.588347809 | 0.000181456 |
| *Gemmatimonadaceae* | 463.0262448 | 2.071076321 | 0.535193797 | 0.000453911 |
| *Acidimicrobiaceae* | 366.6391772 | 1.760321921 | 0.508212808 | 0.001886839 |
| *Micromonosporaceae* | 152.0272389 | 1.923264409 | 0.628460704 | 0.005528292 |
| *Solirubrobacteraceae* | 114.7633207 | 1.91416848 | 0.571306195 | 0.002444309 |
| *Intrasporangiaceae* | 111.1246338 | 2.435159256 | 0.928676387 | 0.016801645 |
| *lfsSE= log2 Fold Change Standard Error | | | | |

| Supplementary Table 11. DESeq2 outputs stem elongation field grown- Significantly differentially abundant bacterial taxa | | | | |
| --- | --- | --- | --- | --- |
| **Comparison - Bulk Soil-Rhizosphere** | | | | |
| **OTU** | **Base Mean** | **log2 Fold Change** | **lfcSE *** | **padj** |
| *Bacillaceae* | 372.2125 | -0.14622 | 0.239322 | 0.693852 |
| *Bacteriovoracaceae* | 13.98168 | 2.230435 | 0.619679 | 0.002832 |
| *Burkholderiaceae* | 1021.925 | 0.813465 | 0.288343 | 0.029905 |
| *Caulobacteraceae* | 110.498 | 1.628402 | 0.494308 | 0.00759 |
| *Chitinophagaceae* | 165.0146 | 0.94339 | 0.293433 | 0.009317 |
| *Devosiaceae* | 101.8837 | 2.40246 | 0.519061 | 0.000123 |
| *Gaiellaceae* | 258.2943 | -0.38662 | 0.113536 | 0.006009 |
| *Geminicoccaceae* | 26.37856 | -0.35258 | 0.514868 | 0.640873 |
| *Haliangiaceae* | 26.72952 | -0.06375 | 0.511428 | 0.948208 |
| *Hymenobacteraceae* | 24.41687 | -0.34667 | 0.477006 | 0.631582 |
| *Ilumatobacteraceae* | 104.5866 | -0.42953 | 0.354796 | 0.383108 |
| *Microbacteriaceae* | 280.9652 | 0.790671 | 0.218822 | 0.003023 |
| *Micromonosporaceae* | 70.07192 | -0.09121 | 0.283765 | 0.849883 |
| *Mycobacteriaceae* | 72.29347 | 0.013391 | 0.291328 | 0.982999 |
| *Nitrosomonadaceae* | 34.0193 | -0.54395 | 0.5127 | 0.435947 |
| *Nitrospiraceae* | 34.26428 | -1.08858 | 0.331452 | 0.006069 |
| *Opitutaceae* | 11.38435 | 1.069774 | 0.522292 | 0.094273 |
| *Paenibacillaceae* | 373.3526 | 0.511014 | 0.16033 | 0.009575 |
| *Pedosphaeraceae* | 24.25692 | -1.34006 | 0.45871 | 0.016595 |
| *Planococcaceae* | 52.96895 | -0.19828 | 0.286047 | 0.640873 |
| *Promicromonosporaceae* | 141.5928 | 1.937938 | 0.499305 | 0.001299 |
| *Pseudomonadaceae* | 400.3107 | 1.768233 | 0.417393 | 0.000454 |
| *Pseudonocardiaceae* | 34.84908 | -0.24604 | 0.541155 | 0.773224 |
| *Rhizobiaceae* | 393.09 | 1.211811 | 0.182278 | 2.97E-09 |
| *Roseiflexaceae* | 108.7535 | 0.144605 | 0.398015 | 0.823412 |
| *Rubritaleaceae* | 100.1947 | -0.89572 | 0.244092 | 0.002699 |
| Saccharimonadaceae | 26.26498 | 1.690537 | 0.510286 | 0.00759 |
| Solirubrobacteraceae | 129.6696 | -0.37406 | 0.141224 | 0.044888 |
| Sphingobacteriaceae | 776.3987 | 1.982141 | 0.452171 | 0.000292 |
| Spirosomaceae | 257.6693 | 2.757245 | 0.49784 | 1.53E-06 |
| Streptomycetaceae | 380.0181 | 0.757702 | 0.323487 | 0.076663 |
| Xanthobacteraceae | 252.9046 | -0.04345 | 0.295926 | 0.939636 |
| **Comparison – Rhizosphere-Endosphere** | | | | |
| **OTU** | **Base Mean** | **log2 Fold Change** | **lfcSE*** | **padj** |
| Bacillaceae | 372.2125 | -0.14622 | 0.239322 | 0.693852 |
| Bacteriovoracaceae | 13.98168 | 2.230435 | 0.619679 | 0.002832 |
| Burkholderiaceae | 2152.24 | 1.142141 | 0.254425 | 0.000102 |
| Caulobacteraceae | 326.3971 | 1.281452 | 0.530015 | 0.053849 |
| Chitinophagaceae | 306.5415 | 0.988075 | 0.277398 | 0.002832 |
| Devosiaceae | 310.9739 | 1.247119 | 0.265459 | 4.38E-05 |
| Gaiellaceae | 65.93289 | -0.57671 | 0.483931 | 0.388951 |
| Geminicoccaceae | 26.37856 | -0.35258 | 0.514868 | 0.640873 |
| Haliangiaceae | 26.72952 | -0.06375 | 0.511428 | 0.948208 |
| Hymenobacteraceae | 24.41687 | -0.34667 | 0.477006 | 0.631582 |
| Ilumatobacteraceae | 104.5866 | -0.42953 | 0.354796 | 0.383108 |
| Microbacteriaceae | 364.1172 | 0.699782 | 0.316859 | 0.068361 |
| Micromonosporaceae | 243.0461 | 1.932803 | 0.296194 | 3.39E-09 |
| Mycobacteriaceae | 72.29347 | 0.013391 | 0.291328 | 0.982999 |
| Nitrosomonadaceae | 34.0193 | -0.54395 | 0.5127 | 0.435947 |
| Nitrospiraceae | 34.26428 | -1.08858 | 0.331452 | 0.006069 |
| Opitutaceae | 11.38435 | 1.069774 | 0.522292 | 0.094273 |
| Paenibacillaceae | 367.5622 | 0.532595 | 0.241368 | 0.068361 |
| Pedosphaeraceae | 24.25692 | -1.34006 | 0.45871 | 0.016595 |
| Planococcaceae | 52.96895 | -0.19828 | 0.286047 | 0.640873 |
| Promicromonosporaceae | 631.1794 | 1.583836 | 0.370707 | 0.000242 |
| Pseudomonadaceae | 337.4026 | -0.05043 | 0.471101 | 0.952873 |
| Pseudonocardiaceae | 314.0513 | 2.733577 | 0.434434 | 7.82E-09 |
| Rhizobiaceae | 503.9776 | 0.568904 | 0.274098 | 0.090323 |
| Roseiflexaceae | 108.7535 | 0.144605 | 0.398015 | 0.823412 |
| Rubritaleaceae | 140.9361 | 0.749244 | 0.331561 | 0.062729 |
| Saccharimonadaceae | 115.8647 | 1.595737 | 0.55365 | 0.017169 |
| Solirubrobacteraceae | 37.03626 | -0.3467 | 0.346116 | 0.465436 |
| Sphingobacteriaceae | 1388.722 | 0.797741 | 0.338137 | 0.054036 |
| Spirosomaceae | 607.5499 | 1.017229 | 0.414429 | 0.052248 |
| Streptomycetaceae | 4613.887 | 2.536194 | 0.400799 | 7.82E-09 |
| Xanthobacteraceae | 252.9046 | -0.04345 | 0.295926 | 0.939636 |
| *lfsSE= log2 Fold Change Standard Error | | | | |

| Supplementary Table 12. DESeq2 outputs (all pot grown and stem elongation)- Significantly differentially abundant bacteria | | | | |
| --- | --- | --- | --- | --- |
| **Comparison - Bulk Soil-Rhizosphere** | | | | |
| **OTU** | **Base Mean** | **log2 Fold Change** | **lfcSE *** | **padj** |
| Unknowns | 2068.626 | -1.19715 | 0.411885 | 0.026106 |
| *Burkholderiaceae* | 985.327 | 3.179403 | 0.485224 | 2.83E-09 |
| Uncultured | 756.9371 | -1.25519 | 0.465714 | 0.046897 |
| Unassigned | 638.8419 | -1.13566 | 0.296593 | 0.001838 |
| *Rhizobiaceae* | 308.3923 | 1.995953 | 0.319026 | 9.85E-09 |
| *Streptomycetaceae* | 292.7344 | 1.160709 | 0.344891 | 0.008221 |
| *Pseudomonadaceae* | 267.3359 | 4.65929 | 0.654589 | 1.10E-10 |
| *Rubritaleaceae* | 184.9994 | 3.54871 | 0.808995 | 0.00023 |
| env.OPS 17 | 136.8828 | -2.13424 | 0.673093 | 0.012669 |
| *Haliangiaceae* | 80.43715 | -0.94404 | 0.278696 | 0.008221 |
| *Spirosomaceae* | 59.57213 | 4.777798 | 0.742664 | 4.16E-09 |
| *Pyrinomonadaceae* | 58.62283 | -2.1681 | 0.648117 | 0.008221 |
| *Fibrobacteraceae* | 57.28673 | 2.684165 | 0.854304 | 0.012911 |
| metagenome | 54.20017 | -1.47081 | 0.382967 | 0.001838 |
| *Cellvibrionaceae* | 25.5762 | 2.138713 | 0.668686 | 0.012564 |
| **Comparison – Rhizosphere-Endosphere** | | | | |
| **OTU** | **Base Mean** | **log2 Fold Change** | **lfcSE*** | **padj** |
| *Streptomycetaceae* | 1540.243 | 4.577 | 0.532667 | 7.14E-16 |
| *Erysipelotrichaceae* | 7.280075 | 6.11435 | 0.737884 | 4.91E-15 |
| *Thermomonosporaceae* | 14.85748 | 4.76837 | 0.696907 | 2.18E-10 |
| *Pseudonocardiaceae* | 88.20983 | 4.75912 | 0.809506 | 8.67E-08 |
| *Pedosphaeraceae* | 14.18555 | -2.540781 | 0.498724 | 5.87E-06 |
| *Polyangiaceae* | 161.9785 | 3.4803 | 0.769874 | 8.63E-05 |
| *Chitinophagaceae* | 424.247 | 3.02177 | 0.705147 | 0.000192 |
| *Rhizobiaceae* | 267.3061 | 1.11239 | 0.258018 | 0.000192 |
| *Promicromonosporaceae* | 88.51291 | 3.44988 | 0.844883 | 0.000414 |
| Unassigned | 78.08997 | -1.719875 | 0.424449 | 0.000427 |
| *Gemmatimonadaceae* | 70.10253 | -2.198493 | 0.561945 | 0.000698 |
| *Solirubrobacteraceae* | 19.04678 | -1.762621 | 0.490947 | 0.002313 |
| *Paenibacillaceae* | 123.1825 | 2.2608 | 0.650672 | 0.003306 |
| WD2101 soil group | 30.91117 | -2.731383 | 0.849034 | 0.007771 |
| *Cellvibrionaceae* | 27.81366 | 2.00047 | 0.628252 | 0.008129 |
| *Cytophagaceae* | 14.87105 | 1.99234 | 0.644695 | 0.010495 |
| *Microscillaceae* | 152.3535 | 2.09948 | 0.719648 | 0.017442 |
| uncultured actinobacterium | 8.533708 | -1.928468 | 0.684966 | 0.022733 |
| *Saccharimonadaceae* | 21.08344 | 2.26986 | 0.819863 | 0.02489 |
| CPla-3 termite group | 11.36798 | -2.312846 | 0.905758 | 0.044792 |
| *lfsSE= log2 Fold Change Standard Error | | | | |

| Supplementary Table 13. DESeq2 outputs senescent plants- Significantly differentially abundant taxa | | | | |
| --- | --- | --- | --- | --- |
| **Comparison - Bulk Soil-Rhizosphere** | | | | |
| **OTU** | **Base Mean** | **log2 Fold Change** | **lfcSE*** | **padj** |
| *Bacillaceae* | 767.3759 | 0.075595 | 0.050122 | 0.26299 |
| *Bacteriovoracaceae* | 355.3372 | 0.446428 | 0.06923 | 1.13E-08 |
| *Burkholderiaceae* | 1720.065 | 0.178619 | 0.035847 | 1.25E-05 |
| *Caulobacteraceae* | 161.8836 | -0.19486 | 0.11932 | 0.227669 |
| *Chitinophagaceae* | 2062.64 | 0.028955 | 0.034216 | 0.521632 |
| *Devosiaceae* | 91.78086 | 0.204319 | 0.204316 | 0.466624 |
| *Gaiellaceae* | 390.196 | 0.238407 | 0.092834 | 0.032987 |
| *Geminicoccaceae* | 283.8183 | 0.288585 | 0.099276 | 0.01521 |
| *Gemmataceae* | 304.0407 | 0.205954 | 0.083663 | 0.043212 |
| *Haliangiaceae* | 430.3705 | 0.249691 | 0.071967 | 0.003725 |
| *Ilumatobacteraceae* | 576.283 | 0.137523 | 0.072957 | 0.156406 |
| *Microbacteriaceae* | 235.302 | 0.068233 | 0.097531 | 0.576395 |
| *Micromonosporaceae* | 237.3228 | 0.059733 | 0.090561 | 0.592464 |
| *Mycobacteriaceae* | 211.5452 | -0.04304 | 0.122052 | 0.775018 |
| *Nitrosomonadaceae* | 631.786 | 0.202049 | 0.060474 | 0.005216 |
| *Nitrospiraceae* | 348.0111 | 0.019542 | 0.069559 | 0.812289 |
| *Opitutaceae* | 516.4374 | -0.19252 | 0.062091 | 0.010162 |
| *Paenibacillaceae* | 486.8574 | -0.28646 | 0.072825 | 0.000837 |
| *Pedosphaeraceae* | 445.4718 | 0.147492 | 0.083526 | 0.184347 |
| *Planococcaceae* | 347.0514 | -0.26987 | 0.101567 | 0.027178 |
| *Pseudomonadaceae* | 166.4812 | 0.286513 | 0.147584 | 0.142084 |
| *Pseudonocardiaceae* | 103.1054 | -0.48027 | 0.186006 | 0.032744 |
| *Rhizobiaceae* | 540.1628 | 0.190274 | 0.061999 | 0.010739 |
| *Rhodomicrobiaceae* | 222.2028 | 0.566638 | 0.102377 | 1.15E-06 |
| *Roseiflexaceae* | 246.7347 | 0.436395 | 0.133666 | 0.006443 |
| *Rubritaleaceae* | 404.6714 | 0.07716 | 0.106963 | 0.567085 |
| *Saccharimonadaceae* | 214.7912 | 0.164709 | 0.105573 | 0.247343 |
| *Solirubrobacteraceae* | 153.0475 | 0.174746 | 0.163668 | 0.432822 |
| *Sphingobacteriaceae* | 319.3659 | -0.48343 | 0.119487 | 0.000586 |
| *Spirosomaceae* | 163.2731 | -0.09857 | 0.12104 | 0.521632 |
| *Streptomycetaceae* | 189.924 | -0.12651 | 0.11795 | 0.432822 |
| *Xanthobacteraceae* | 360.5323 | -0.25971 | 0.072029 | 0.002396 |
| **Comparison – Rhizosphere-Endosphere** | | | | |
| **OTU** | **Base Mean** | **log2 Fold Change** | **lfcSE*** | **padj** |
| *Bacillaceae* | 669.8586 | -0.24338 | 0.093574 | 0.02817 |
| *Bacteriovoracaceae* | 211.6517 | -0.38527 | 0.16022 | 0.0426 |
| *Burkholderiaceae* | 1560.524 | -0.05437 | 0.071256 | 0.556862 |
| *Caulobacteraceae* | 161.1426 | -0.32272 | 0.179428 | 0.126463 |
| *Chitinophagaceae* | 2810.665 | 0.334696 | 0.09971 | 0.003625 |
| *Gaiellaceae* | 616.729 | 0.668651 | 0.14625 | 5.37E-05 |
| *Geminicoccaceae* | 253.4938 | 0.051528 | 0.157662 | 0.835729 |
| *Gemmataceae* | 609.9269 | 0.873427 | 0.10693 | 1.56E-14 |
| *Haliangiaceae* | 412.6111 | 0.106914 | 0.097998 | 0.393264 |
| *Hymenobacteraceae* | 421.6195 | 0.664185 | 0.119973 | 6.19E-07 |
| *Ilumatobacteraceae* | 1702.794 | 1.175225 | 0.140792 | 6.99E-15 |
| *Microbacteriaceae* | 174.5953 | -0.56967 | 0.190141 | 0.01013 |
| *Micromonosporaceae* | 251.2053 | 0.040195 | 0.145248 | 0.839919 |
| *Mycobacteriaceae* | 430.8715 | 0.710153 | 0.17698 | 0.000429 |
| *Nitrosomonadaceae* | 687.0426 | 0.216746 | 0.141276 | 0.19838 |
| *Nitrospiraceae* | 613.7027 | 0.610044 | 0.137464 | 8.26E-05 |
| *Opitutaceae* | 1042.123 | 0.607463 | 0.131676 | 4.95E-05 |
| *Paenibacillaceae* | 959.6071 | 0.525368 | 0.079788 | 1.52E-09 |
| *Pedosphaeraceae* | 677.0895 | 0.550655 | 0.095806 | 2.26E-07 |
| *Planococcaceae* | 369.4337 | -0.28308 | 0.191611 | 0.214734 |
| *Pseudomonadaceae* | 195.1522 | 0.386658 | 0.234509 | 0.165314 |
| *Pseudonocardiaceae* | 222.4399 | 0.516128 | 0.159337 | 0.005211 |
| *Rhizobiaceae* | 385.9288 | -0.45277 | 0.114062 | 0.00048 |
| *Rhodomicrobiaceae* | 100.4318 | -0.77128 | 0.17287 | 8.13E-05 |
| *Roseiflexaceae* | 144.6883 | -0.43948 | 0.214584 | 0.08816 |
| *Rubritaleaceae* | 366.9267 | -0.17432 | 0.175362 | 0.444729 |
| *Saccharimonadaceae* | 209.715 | 0.035154 | 0.126667 | 0.839919 |
| *Solirubrobacteraceae* | 140.2452 | -0.04875 | 0.182615 | 0.839919 |
| *Sphingobacteriaceae* | 599.1594 | 0.360183 | 0.106568 | 0.003625 |
| *Spirosomaceae* | 187.2959 | -0.00022 | 0.28912 | 0.999402 |
| *Streptomycetaceae* | 371.4646 | 0.613746 | 0.142608 | 0.00014 |
| *Xanthobacteraceae* | 370.2052 | -0.33994 | 0.144082 | 0.04694 |
| *lfsSE= log2 Fold Change Standard Error | | | | |

| Supplementary Table 14. DESeq2 outputs stem elongation/senescent plants- Significantly differentially abundant bacterial taxa | | | | |
| --- | --- | --- | --- | --- |
| **Comparison – Senescent endosphere/Stem elongation endosphere** | | | | |
| **OTU** | **Base Mean** | **log2 Fold Change** | **lfcSE*** | **padj** |
| *Bacillaceae* | 357.5098 | -0.81595 | 0.241208 | 0.004485 |
| *Bacteriovoracaceae* | 25.6759 | -1.66011 | 0.594842 | 0.019471 |
| *Burkholderiaceae* | 3318.41 | -1.25399 | 0.238716 | 1.66E-06 |
| *Caulobacteraceae* | 541.1063 | -0.79572 | 0.360076 | 0.072479 |
| *Chitinophagaceae* | 870.3272 | 0.176643 | 0.254357 | 0.587213 |
| *Devosiaceae* | 528.9238 | -1.11192 | 0.28085 | 0.000579 |
| *Gaiellaceae* | 68.69234 | 0.214398 | 0.45143 | 0.69004 |
| *Geminicoccaceae* | 38.29272 | 0.370222 | 0.423023 | 0.515508 |
| *Haliangiaceae* | 47.10503 | 0.248007 | 0.470804 | 0.657529 |
| *Hymenobacteraceae* | 19.25489 | -0.84456 | 0.475021 | 0.157109 |
| *Ilumatobacteraceae* | 160.0597 | 0.364665 | 0.301336 | 0.395293 |
| *Microbacteriaceae* | 656.3924 | -0.31577 | 0.245576 | 0.354469 |
| *Micromonosporaceae* | 1086.754 | -1.17346 | 0.357779 | 0.005467 |
| *Mycobacteriaceae* | 84.1459 | -0.47063 | 0.259804 | 0.149081 |
| *Nitrosomonadaceae* | 43.46863 | 0.355034 | 0.473786 | 0.567054 |
| *Nitrospiraceae* | 33.82162 | 0.659862 | 0.322814 | 0.090987 |
| *Opitutaceae* | 22.22379 | -0.56716 | 0.489488 | 0.407432 |
| *Paenibacillaceae* | 431.831 | -1.74822 | 0.248606 | 5.09E-11 |
| *Pedosphaeraceae* | 18.52983 | 0.736093 | 0.466216 | 0.219935 |
| *Planococcaceae* | 59.45307 | -0.31161 | 0.280003 | 0.407432 |
| *Promicromonosporaceae* | 1086.754 | -1.17346 | 0.357779 | 0.005467 |
| *Pseudomonadaceae* | 304.2958 | -0.94093 | 0.341331 | 0.020137 |
| *Pseudonocardiaceae* | 878.523 | -0.10126 | 0.262648 | 0.73668 |
| *Rhizobiaceae* | 1302.42 | 0.255903 | 0.231463 | 0.407432 |
| *Rhodomicrobiaceae* | 5.325793 | 0.713473 | 0.635217 | 0.407432 |
| *Roseiflexaceae* | 104.1034 | -1.21258 | 0.403322 | 0.010405 |
| *Rubritaleaceae* | 238.5271 | -0.68881 | 0.318014 | 0.075784 |
| *Saccharimonadaceae* | 613.9465 | 0.60443 | 0.56084 | 0.41666 |
| *Solirubrobacteraceae* | 40.43118 | -0.13417 | 0.411122 | 0.775156 |
| *Sphingobacteriaceae* | 1977.09 | -1.36091 | 0.330803 | 0.000324 |
| *Spirosomaceae* | 815.2618 | -1.9214 | 0.301822 | 3.23E-09 |
| *Streptomycetaceae* | 7007.769 | -2.15252 | 0.314852 | 1.62E-10 |
| *Xanthobacteraceae* | 424.1464 | 0.19374 | 0.240251 | 0.554194 |
| *lfsSE= log2 Fold Change Standard Error | | | | |

| Supplementary Table 15. DESeq2 outputs Significantly differentially abundant fungal taxa 01 | | | | |
| --- | --- | --- | --- | --- |
| **Comparison - Bulk Soil-Rhizosphere Senescent** | | | | |
| **OTU** | **Base Mean** | **log2 Fold Change** | **lfcSE*** | **padj** |
| *Ambisporaceae* | 136.0381 | -7.31509 | 1.504427 | 3.60E-05 |
| *Chaetosphaeriaceae* | 583.7226 | -4.90418 | 1.272226 | 0.000891 |
| *Cladosporiaceae* | 1751.722 | 0.352167 | 0.865945 | 0.861533 |
| *Erythrobasidiaceae* | 88.04139 | -0.04082 | 0.908122 | 0.964151 |
| *Hypocreales Incertae sedis* | 11149.53 | -0.45642 | 0.716685 | 0.774593 |
| *Mortierellaceae* | 3301.221 | -2.72388 | 0.842895 | 0.004771 |
| *Sporidiobolaceae* | 535.6329 | 3.439894 | 0.856549 | 0.000612 |
| *Sydowiellaceae* | 524.9519 | -0.50637 | 0.762741 | 0.774593 |
| *Parmeliaceae* | 40.84577 | 5.768819 | 1.221575 | 3.61E-05 |
| **Comparison – Rhizosphere-Endosphere Senescent** | | | | |
| **OTU** | **Base Mean** | **log2 Fold Change** | **lfcSE*** | **padj** |
| *Ambisporaceae* | 1.053667 | 1.051434 | 2.149638 | 0.756691 |
| *Chaetosphaeriaceae* | 43.17347 | 2.16618 | 0.73166 | 0.019034 |
| *Cladosporiaceae* | 438.7817 | -1.79186 | 0.722699 | 0.049133 |
| *Erythrobasidiaceae* | 19.80491 | -4.76126 | 1.234989 | 0.001089 |
| *Hypocreales Incertae sedis* | 2296.368 | -2.83945 | 0.668941 | 0.000339 |
| *Mortierellaceae* | 225.3781 | -1.99924 | 0.697942 | 0.021581 |
| *Parmeliaceae* | 15.73176 | 1.890016 | 1.11563 | 0.233124 |
| *Sporidiobolaceae* | 191.0621 | -3.80797 | 0.717967 | 3.52E-06 |
| *Sydowiellaceae* | 117.4911 | -1.88265 | 0.696616 | 0.030472 |
| **Comparison – Bulk Soil-Rhizosphere Stem Elongation** | | | | |
| *Ambisporaceae* | 1.126446 | 0.297184 | 1.528897 | 0.895664 |
| *Chaetosphaeriaceae* | 17.49084 | -0.70025 | 1.581208 | 0.895664 |
| *Cladosporiaceae* | 499.0058 | -0.33263 | 0.773925 | 0.895664 |
| *Erythrobasidiaceae* | 266.2979 | 2.846739 | 1.621902 | 0.369093 |
| *Hypocreales Incertae sedis* | 8407.459 | -1.26002 | 0.733516 | 0.369093 |
| *Mortierellaceae* | 3348.64 | 3.034097 | 0.857525 | 0.017323 |
| *Sporidiobolaceae* | 979.9934 | 0.496755 | 0.954706 | 0.895664 |
| *Sydowiellaceae* | 810.7476 | -1.01088 | 0.795848 | 0.461721 |
| *Parmeliaceae* | 946.0702 | 0.948964 | 0.83265 | 0.497266 |
| **Comparison – Rhizosphere-Endosphere Stem Elongation** | | | | |
| *Ambisporaceae* | 3.5 | 2.584955 | 1.466257 | 0.136337 |
| *Chaetosphaeriaceae* | 6.666667 | -2.23704 | 1.439501 | 0.168246 |
| *Cladosporiaceae* | 211.6667 | -3.02444 | 1.295549 | 0.053817 |
| *Erythrobasidiaceae* | 191.5 | -5.43269 | 1.6628 | 0.004345 |
| *Hypocreales Incertae sedis* | 2356.833 | -2.30279 | 0.561104 | 0.000314 |
| *Mortierellaceae* | 2594.5 | -6.9257 | 0.786027 | 3.47E-17 |
| *Parmeliaceae* | 2737.5 | 2.135562 | 0.52331 | 0.000314 |
| *Sporidiobolaceae* | 525.3333 | -6.51894 | 1.131744 | 1.18E-07 |
| *Sydowiellaceae* | 239.1667 | -3.20945 | 0.839198 | 0.000734 |
| *lfsSE= log2 Fold Change Standard Error | | | | |

| Supplementary Table 16. DESeq2 outputs Significantly differentially abundant fungal taxa 02 | | | | |
| --- | --- | --- | --- | --- |
| **Comparison - Endosphere Senescent-Endosphere Stem Elongation** | | | | |
| **OTU** | **Base Mean** | **log2 Fold Change** | **lfcSE*** | **padj** |
| *Parmeliaceae* | 2232.5 | -9.80089 | 0.980524 | 4.94E-22 |
| *Leotiaceae* | 2225.5 | -12.1196 | 1.281843 | 5.02E-20 |
| *Myxotrichaceae* | 524.5 | -7.1502 | 1.058346 | 1.47E-10 |
| *Clavicipitaceae* | 4820.833333 | -3.95072 | 1.096925 | 0.0014004 |
| *Chaetosphaeriaceae* | 26.66666667 | 4.450025 | 1.070374 | 0.0001663 |
| **Comparison – Bulk Soil-Rhizosphere all samples (compost, agricultural pot, 50:50 mix and field stem elongation)** | | | | |
| **OTU** | **Base Mean** | **log2 Fold Change** | **lfcSE*** | **padj** |
| *Australiascaceae* | 736.0431973 | -0.22788 | 0.803202 | 0.8926117 |
| *Glomerellaceae* | 2480.256112 | -0.23093 | 0.96327 | 0.8926117 |
| *Hypocreales Incertae sedis* | 6355.970558 | -1.04999 | 0.731854 | 0.509278 |
| *Leotiaceae* | 1357.387391 | -1.72508 | 0.760808 | 0.2009295 |
| *Mortierellaceae* | 1382.193766 | 3.010589 | 0.87225 | 0.0119848 |
| **Comparison – Rhizosphere-Endosphere all samples (compost, agricultural pot, 50:50 mix and field stem elongation)** | | | | |
| *Australiascaceae* | 616.0318099 | -3.69862 | 0.722446 | 6.58E-06 |
| *Glomerellaceae* | 2071.880172 | -3.3994 | 0.928719 | 0.0009847 |
| *Hypocreales Incertae sedis* | 3357.081144 | -3.56054 | 0.733033 | 1.59E-05 |
| *Leotiaceae* | 1491.808491 | 3.60561 | 0.749014 | 1.59E-05 |
| *Mortierellaceae* | 1463.011582 | -3.95723 | 1.01644 | 0.0004576 |
| *lfsSE= log2 Fold Change Standard Error | | | | |

**References**

[1] Muyzer G, de Waal EC, Uitterlinden AG. Profiling of complex microbial populations by denaturing gradient gel electrophoresis analysis of polymerase chain reaction-amplified genes coding for 16S rRNA. Applied and Environmental Microbiology 1993;59:695–700. https://doi.org/10.1128/AEM.59.3.695-700.1993.

[2] Ochsenreiter T, Selezi D, Quaiser A, Bonch-Osmolovskaya L, Schleper C. Diversity and abundance of Crenarchaeota in terrestrial habitats studied by 16S RNA surveys and real time PCR. Environ Microbiol 2003;5:787–97. https://doi.org/10.1046/j.1462-2920.2003.00476.x.

[3] Großkopf R, Janssen PH, Liesack W. Diversity and structure of the methanogenic community in anoxic rice paddy soil microcosms as examined by cultivation and direct 16S rRNA gene sequence retrieval. Appl Environ Microbiol 1998;64:960–9. https://doi.org/10.1128/AEM.64.3.960-969.1998.

[4] Gantner S, Andersson AF, Alonso-Sáez L, Bertilsson S. Novel primers for 16S rRNA-based archaeal community analyses in environmental samples. Journal of Microbiological Methods 2011;84:12–8. https://doi.org/10.1016/j.mimet.2010.10.001.

[5] Yu Y, Lee C, Kim J, Hwang S. Group-specific primer and probe sets to detect methanogenic communities using quantitative real-time polymerase chain reaction. Biotechnol Bioeng 2005;89:670–9. https://doi.org/10.1002/bit.20347.

[6] Ihrmark K, Bödeker ITM, Cruz-Martinez K, Friberg H, Kubartova A, Schenck J, et al. New primers to amplify the fungal ITS2 region - evaluation by 454-sequencing of artificial and natural communities. FEMS Microbiol Ecol 2012;82:666–77. https://doi.org/10.1111/j.1574-6941.2012.01437.x.

[7] White TJ, Bruns T, Lee S, Taylor J. Amplification and direct sequencing of fungal ribosomal RNA genes for phylogenetics. PCR Protocols, Elsevier; 1990, p. 315–22. https://doi.org/10.1016/B978-0-12-372180-8.50042-1.

[8] Vainio EJ, Hantula J. Direct analysis of wood-inhabiting fungi using denaturing gradient gel electrophoresis of ampliﬁed ribosomal DNA n.d.:10.

[9] Fredriksson NJ, Hermansson M, Wilén B-M. The choice of PCR primers has great impact on assessments of bacterial community diversity and dynamics in a wastewater treatment plant. PLOS ONE 2013;8:20.

[10] Rastogi G. A PCR-based toolbox for the culture-independent quantification of total bacterial abundances in plant environments. Journal of Microbiological Methods 2010:6.

[11] Takai K, Horikoshi K. Rapid detection and quantification of members of the archaeal community by quantitative PCR using fluorogenic probes. Appl Environ Microbiol 2000;66:5066–72. https://doi.org/10.1128/AEM.66.11.5066-5072.2000.

[12] Tourna M, Freitag TE, Nicol GW, Prosser JI. Growth, activity and temperature responses of ammonia‐oxidizing archaea and bacteria in soil microcosms. Environmental Microbiology 2008:9.
